# Genome-Wide Association Study Identifies *ZNF354C* Variants Associated with Depression from Interferon-Based Therapy for Chronic Hepatitis C

**DOI:** 10.1371/journal.pone.0164418

**Published:** 2016-10-10

**Authors:** Kayoko Matsunami, Nao Nishida, Naoko Kaneko, Kazuho Ikeo, Licht Toyo-oka, Hiroshi Takeuchi, Kentaro Matsuura, Akihiro Tamori, Hideyuki Nomura, Hitoshi Yoshiji, Masatoshi Imamura, Naohiko Masaki, Tatsuro Hayakawa, Tatsuya Ide, Noritomo Shimada, Fusao Ikeda, Keisuke Hino, Shuhei Nishiguchi, Chiaki Okuse, Shunsuke Nojiri, Kazunobu Sawamoto, Katsushi Tokunaga, Takashi Joh, Yasuhito Tanaka

**Affiliations:** 1 Department of Virology & Liver Unit, Nagoya City University Graduate School of Medical Sciences, Nagoya, Japan; 2 Department of Gastroenterology and Metabolism, Nagoya City University Graduate School of Medical Sciences, Nagoya, Japan; 3 The Research Center for Hepatitis and Immunology, National Center for Global Health and Medicine, Ichikawa, Japan; 4 Department of Human Genetics, Graduate School of Medicine, The University of Tokyo, Tokyo, Japan; 5 Department of Developmental and Regenerative Biology, Nagoya City University Graduate School of Medical Sciences, Nagoya, Japan; 6 Laboratory for DNA Data Analysis Center for Information Biology and DNA Data Bank of Japan, National Institute of Genetics, Mishima, Japan; 7 Department of Psychiatry, Japanese Red Cross Nagoya Daini Hospital, Nagoya, Japan; 8 Department of Hepatology, Osaka City University Graduate School of Medicine, Osaka, Japan; 9 The Center for Liver Disease, Shin-Kokura Hospital, Kitakyushu, Japan; 10 Third Department of Internal Medicine, Nara Medical University, Kashihara, Japan; 11 Kohnodai Hospital, National Center for Global Health and Medicine, Ichikawa, Japan; 12 Division of Gastroenterology, Department of Medicine, Kurume University, Kurume, Japan; 13 Division of Gastroenterology and Hepatology, Ootakanomori Hospital, Kashiwa, Japan; 14 Department of Gastroenterology and Hepatology, Okayama University Graduate School of Medicine, Dentistry, and Pharmaceutical Sciences, Okayama, Japan; 15 Division of Hepatology and Pancreatology, Kawasaki Medical College, Kurashiki, Japan; 16 Department of Internal Medicine, Hyogo College of Medicine, Nishinomiya, Japan; 17 Department of Gastroenterology and Hepatology, St. Marianna University School of Medicine, Kawasaki, Japan; National Taiwan University Hospital, TAIWAN

## Abstract

The therapeutic use of interferon (IFN) is known to cause depression that frequently interrupts treatment. To identify genetic variants associated with IFN-induced depression, we conducted a genome-wide association study (GWAS) of 224 Japanese chronic hepatitis C patients receiving IFN-based therapy in a multicenter prospective study and stratified them into two groups according to the Beck Depression Inventory, Second Edition (BDI-II) score. In the GWAS stage, we selected 42 candidate single nucleotide polymorphisms (SNPs) to perform replication analysis in an independent set of 160 subjects. The SNP rs1863918 in strong linkage disequilibrium with SNPs located around the Zinc finger 354C (*ZNF354C*) gene on chromosome 5 showed a significant association when the results of GWAS and replication were combined (odds ratio = 2.55, *P* = 7.89×10^−8^ in the allele frequency model), suggesting that the rs1863918 T allele was associated with IFN-induced depression. Furthermore, logistic regression analysis showed that rs1863918 T allele, a history of depression, and younger age were independent predictive factors for IFN-induced depression. Interestingly, western blotting and immunofluorescence showed that ZNF354C was highly expressed in the hippocampus in mice, a region implicated in the pathology of psychiatric symptoms. In conclusion, we identified rs1863918 as significantly associated with IFN-induced depression, and revealed that the candidate gene *ZNF354C* is highly expressed in the hippocampus of mice. Our data might be useful for elucidating the pathogenic mechanisms of depression induced by drugs including IFN.

## Introduction

Chronic hepatitis C virus (HCV) infection presents a global health problem with a prevalence of more than 130–150 million people. Overall, 55–85% of HCV-infected individuals go on to develop chronic infection, and are at significant risk for progressive liver fibrosis and subsequent liver cirrhosis as well as hepatocellular carcinomas (HCC). Furthermore, over 500,000 people die every year from hepatitis C-related liver diseases [1].

Antiviral treatment has been shown to improve liver histology and decrease the incidence of HCC in chronic hepatitis C (CHC) [2]. The mainstay for CHC treatment was combination therapy using pegylated interferon-α (PEG-IFN) plus ribavirin (RBV) when we started this study; however, only about 50% of these treated patients infected with HCV genotype 1 achieve a sustained virological response (SVR) [3]. Current therapy for CHC consists of direct-acting antivirals combined with PEG-IFN plus RBV, and IFN-free therapies. Thus, more than 80% of patients infected with HCV genotype 1 are reported to achieve SVR [4]. Especially, IFN-free therapies with high SVR are expected to be useful for IFN-ineligible/intolerant patients, and become the standard of care in developed countries [5]. However, as the cost of IFN-free therapies is relatively high, IFN-based regimens are still the standard of care in Asian countries where most patients have *IFNL3-IFNL4* favorable genotypes [6].

IFN-based therapies are associated with various adverse events, such as flu-like syndrome, hematologic abnormalities and adverse neuropsychiatric events [7]. These often necessitate dose reduction and premature withdrawal from IFN-based therapy, resulting in poor treatment efficiency [8]. Depression is a common (15–45%) side-effect of IFN-α treatment and might compromise the effectiveness of therapy [9].

It was reported in previous studies that single nucleotide polymorphisms (SNPs) in inflammation-associated genes such as IFN-α receptor (*IFNAR1*) [10], IL-6 (*IL6*) [11] and IFN-γ (*IFNG*) [12], as well as serotonin-associated genes such as 5-HT transporter (5-HTT) gene transcription initiation site (5-HTT-linked polymorphic region, 5-HTTLPR) [11, 13], 5-HT1A receptor (*HTR1A*) [14], and tryptophan hydroxylase-2 (*TPH2*) [15] are related to the onset of IFN-induced depression. However, little is known about the host genetic factors that might be associated with IFN-induced depression at the level of genome-wide significance by a genome-wide association study (GWAS). Therefore, to identify genetic variants associated with IFN-induced depression, we conducted a GWAS in Japanese CHC patients receiving IFN-based therapy.

## Materials and Methods

### Ethics Statement

The human genome study (No. 67) and BDI-II monitoring (No.417) were approved by Nagoya City University Human Genome Review Committees and were conducted in accordance with the ethical guidelines of the Helsinki Declaration. Written informed consent was obtained from all individual participants included in the study. All experiments using live animals were performed in accordance with the guidelines and regulations of Nagoya City University Institutional Animal Care and Use Committee (H21M-36 and H25M-57).

### Patients

From 2009 to 2012, samples for the GWAS were obtained from 224 Japanese CHC patients who were treated at 11 multi-center hospitals (liver units with hepatologists) throughout Japan. In the following stage of replication analysis, 160 samples were collected from an independent set of Japanese CHC patients. Most patients were treated with PEG-IFN-α2b (1.5 μg/kg body weight subcutaneously once a week) or PEG-IFN-α2a (180 μg once a week) plus RBV (600–1000 mg daily according to body weight), for 48 weeks for HCV genotype 1 and 24 weeks for genotype 2. Treatment duration was extended in some patients up to 72 weeks for genotype 1 and 48 weeks for genotype 2, according to the physicians’ preferences. Other patients were treated with PEG-IFN-α2a monotherapy or IFN-β plus RBV in standard doses of the regimens. The doses of drugs were reduced according to the recommendations on the package inserts or the clinical conditions of the individual patients.

Patients chronically infected with hepatitis B virus or human immunodeficiency virus, or with other causes of liver disease such as autoimmune hepatitis and primary biliary cirrhosis, were excluded from this study.

### The Beck Depression Inventory-II

We enrolled 384 Japanese CHC patients receiving IFN-based therapy in a multicenter prospective study and stratified them into two groups: cases (BDI-II ≥ 20) and controls (BDI-II < 20) according to the Beck Depression Inventory, Second Edition (BDI-II) score at weeks 4, 12, or 24 during IFN-based therapy, and at 12 weeks after the end of therapy [16–18]. Patients with BDI-II ≥ 20 at baseline were excluded from the study before it started. The case group included patients with BDI-II ≥ 20 at some point during the course of therapy, and the control group included patients with BDI-II < 20 at all timepoints.

The BDI-II is one of the most widely used psychometric tests for measuring the severity of depression by health care professionals and researchers in a variety of settings and is a 21-item self-report questionnaire revised to correspond to the diagnostic criteria of the Diagnostic and Statistical Manual of Mental Disorders, Fourth Edition (DSM-IV) published by the American Psychiatric Association [19, 20].

### SNP genotyping and data cleaning

We performed genome-wide SNP typing using the Affymetrix Genome-Wide Human SNP Array 6.0 (Affymetrix, Inc. Santa Clara, CA) for 900,000 SNPs according to the manufacturer's instructions. The cut-off value was calculated to maximize the difference, which was also close to the median change. All samples had a quality control (QC) call rate over 0.95 and overall call rate for a total of 900K SNPs over 0.96. No samples were excluded by heterozygosity check and identity by descent (IBD) check. Furthermore, population outliers were not identified based on the principal component analysis (PCA). After data cleaning, we performed GWAS using genotype calls that were determined together with cases and controls. Quantile-quantile (QQ) plots were generated on the basis of an allele-wise analysis of SNPs that passed the QC criteria. At GWAS, the average overall call rate of patients in the case and the control group reached 99.18% (97.00–99.60%) and 99.26% (98.08–99.68%), respectively. We then applied the following thresholds for SNP QC in SNP filtering: SNP call rate ≥ 95%, minor allele frequency (MAF) ≥ 5%, and Hardy-Weinberg equilibrium (HWE) *P*-value ≥ 0.001. A total of 551,176 SNPs on autosomal chromosomes passed QC filters and were used in the genome-wide association analysis. Statistical analyses for GWAS were performed using the SNP & Variation Suite software (Golden Helix, Bozeman, MT).

All cluster plots of SNPs showing *P*<10^−4^ in association analyses by comparing allele frequencies in both groups were checked by visual inspection and 4 SNPs with ambiguous genotype calls were excluded. In the replication study, the genotyping of 42 candidate SNPs in an independent set of 160 Japanese HCV-infected patients was performed using the DigiTag2 assay [21] and TaqMan SNP Genotyping Assays (Applied Biosystems, Foster City, CA) on the LightCycler 480 Real-Time PCR System (Roche, Mannheim, Germany). Samples for the replication study were obtained in the same way as for the GWAS samples set. All 42 genotyped SNPs in the replication analysis had a >95% call rate, and cleared HWE *P*≥0.001.

### SNP Imputation

Unobserved genotypes were imputed using the phased genotype data of 1000 Genomes Project reference data (Integrated Phase 3, June 2014 released) with standard software packages such as IMPUTE2 version 2 [22] with default parameters. GTOOL was used for the data format conversion from PLINK to IMPUTE2. A 1-Mb window size centered on each candidate SNPs was applied to impute. After imputation, the results of an association test for imputed data were obtained using PLINK 1.07. SNPs with >1% missing genotype data, HWE *P*-value≥0.001, and samples including >10% missing genotypes were eliminated.

### Laboratory and histological tests

Blood samples were obtained at baseline and at appropriate periods after the start of therapy and for hematologic tests, blood chemistry, and HCV RNA. A genetic polymorphism in the *IFNL3-IFNL4* gene (rs8099917) was determined using the ABI TaqMan SNP assays. Fibrosis was evaluated on a scale of 0–4 according to the METAVIR scoring system. The SVR was defined as an undetectable HCV RNA level by Roche COBAS Amplicor HCV Monitor test, v.2.0 (Roche Molecular Diagnostics, Pleasanton, CA) with a lower detection limit of 50 IU/ml or Roche COBAS AmpliPrep/COBAS TaqMan HCV assay (Roche Molecular Diagnostics, Pleasanton, CA) with a lower detection limit of 15 IU/ml at 24 weeks after completion of the therapy.

### Statistical analysis

The observed association between a SNP and depression induced by IFN-based therapy was assessed by the chi-square test with a two-by-two contingency table in the allele frequency model. The standard criterion for significance in GWAS was *P*<5.00×10^−8^. Significance levels after Bonferroni correction for multiple testing were *P* = 9.07×10^−8^ (0.05/551,176) in the GWAS stage and *P* = 0.0012 (0.05/42) in the replication stage.

Categorical variables were compared between groups by the chi-square test, and non-categorical variables by the Mann-Whitney *U*-test. Multivariate logistic regression analysis with stepwise forward selection was performed: inclusion of explanatory variables in the models was based on significant factors by univariate analysis and existing knowledge of risk factors for IFN-induced depression. Statistical analyses were performed using SPSS version 19 for windows (SPSS, Chicago, Illinois, USA). *P*<0.05 was considered significant in all tests.

### Animal experiments

#### Administration of IFN-α to mice

Male, 8-week-old C57BL/6J mice were purchased from SLC (Shizuoka, Japan). PBS or mouse IFN-α (4×10^5^ IU/kg, Miltenyi Biotec, Auburn, CA, USA) diluted with PBS was intraperitoneally injected into mice once a day for 4 weeks. Mouse body weights were measured daily throughout the course of treatment.

#### RNA extraction and real-time detection polymerase chain reaction (PCR)

To examine the mRNA levels of the Zinc finger 354C (*ZNF354C*) gene, mice were sacrificed under deep anesthesia by overdose inhalation of isoflurane, and their kidney and brain tissues were collected. Total RNA was extracted from the tissues with TRIzol reagent (Invitrogen, Carlsbad, CA, USA) and cDNA synthesis was performed using the SuperScript First-Strand Synthesis System for RT-PCR (Invitrogen). Quantitative SYBR Green real time PCR was performed as follows. Briefly, each 25 μl of SYBR Green reaction consisted of 5 μl of cDNA (50 ng/μl), 12.5 μl of 2× Universal SYBR Green PCR Master Mix (Applied Biosystems, Carlsbad, CA, USA), and 3.75 μl each of 50 nM forward and reverse primers. Primer sequences were designed using Primer Express Software (Applied Biosystems). Quantitative RT-PCR was performed on an ABI 7500 Fast Real-Time PCR instrument (Applied Biosystems) using the following 3-stage program parameters provided by the manufacturer: 2 min at 50°C, 10 min at 95°C, 40 cycles of 15 s at 95°C, and finally 1 min at 60°C. Each sample was tested in duplicate, and the data are expressed as the fold change in gene expression relative to the PBS control group. The following primers were used: 5′-CCGGCGTCCGCATATTT-3′ and 5′-CCCTTCTTAGTTTTTCTGCCAAAG-3′, which amplify a 58-bp ZNF354C product; and 5′-CATGGCCTTCCGTGTTCCTA-3′ and 5′-CACGTCAGATCCA-3′, which amplify a 55-bp GAPDH product. Commercial primers (Quantitect Primer Assay, Mm_Zfp354c_1_SG, QT00126728, Qiagen, Hilden, Germany) were used to confirm the results.

### Western blotting

Kidney and brain tissues were homogenized in lysis buffer (20 mM Tris—HCl, pH 8.0, 100 mM NaCl, 1 mM EDTA, 10 μg/ml leupeptin, and 10 μM phenylmethylsulfonyl fluoride). Lysates were briefly sonicated and cleared by centrifugation. The samples were separated by SDS-polyacrylamide gel electrophoresis. Proteins were transferred to polyvinylidene difluoride membranes, blocked in 3% skim milk in Tris buffered saline containing 0.01% Tween 20, incubated with primary antibodies (goat anti-ZNF354c, 1:300, Santa Cruz Biotechnology, Santa Cruz, CA, USA; mouse anti-actin, 1:10,000, Merck Millipore, Billerica, MA, USA) and detected by horseradish peroxidase-conjugated secondary antibodies (Dako, Glostrup, Denmark) with enhanced luminal-based chemiluminescent western blotting detection reagent (GE Healthcare, Buckinghamshire, UK). Signals were detected and measured with a cooled charge-coupled device camera (LAS3000mini, Fujifilm, Tokyo, Japan).

### Immunofluorescence and microscopy

Mouse brain sections were prepared and stained as previously described [23]. Briefly, the brains were extracted and post-fixed with 4% paraformaldehyde in 0.1 M phosphate buffer overnight, then cut into 50-μm-thick coronal sections on a vibratome (VT1200S, Leica, Wetzlar, Germany). The sections were incubated for 1 h in blocking solution (10% donkey serum and 0.4% Triton X-100 in PBS), then overnight at 4°C with primary antibodies, and for 2 h at room temperature with Alexa Fluor-conjugated secondary antibodies (1:1000, Life Technology, Carlsbad, CA, USA). The following primary antibodies were used: goat anti-ZNF354C (1:200, Santa Cruz Biotechnology) and mouse anti-NeuN (1:100, Merck Millipore). Nuclei were stained with Hoechst 33342 (1:5000, Thermo Fisher Scientific). Confocal images were obtained using a LSM700 laser-scanning microscope system (Zeiss, Oberkochen, Germany).

## Results

### Genetic variants associated with IFN-induced depression

**Fig 1:**
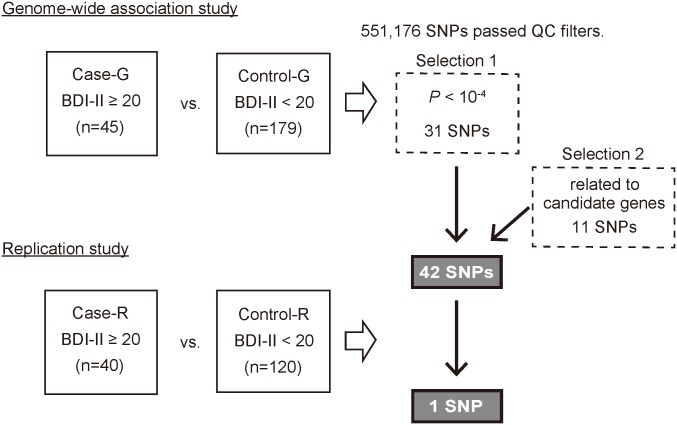
Outline of the study design. BDI-II, Beck Depression Inventory-II; SNP, single nucleotide polymorphism; QC, quality control; OR, odds ratio.

In this study, we conducted GWAS analysis to identify host genes associated with depression in response to IFN-based therapy, followed by replication analysis (Fig 1). The characteristics of patients for each GWAS stage and replication stage are summarized in Table 1. The mean age was significantly lower in the case group than in the control group only at the replication stage (*P*<0.001). The rate of patients with a history of depression was significantly higher in the case group than in the control group at both the GWAS and replication stages (*P*<0.001).

**Table 1:** Clinical characteristics of patients in GWAS and replication study.

	GWAS		Replication	
	Case	Control	Case	Control
	(n = 45)	(n = 179)	(n = 40)	(n = 120)
Age	55.7 (10.7)	57.3 (11.6)	49.9 (11.8)	58.0 (10.6)
Gender (Male/Female)	17/28	88/91	24/16	65/55
Genotype (1/2/N.D.)	26/19/0	113/65/1	26/14/0	93/26/1
Liver fibrosis (F0-2/F3-4/N.D.)	26/4/15	88/33/58	13/4/23	53/18/49
Type of IFN (Peg-IFN-α2a/Peg-IFN-α2b/IFN-β)	11/31/3	55/111/13	4/29/7	12/103/5
Period for administration of IFN (weeks)	39.3 (20.0)	38.3 (18.2)	27.8 (11.5)	29.8 (12.3)
Effect of treatment (rate of SVR, %)	75.6	59.2	82.5	84.2
*IL28B*, rs8099917 (TT/TG+GG/N.D.)	29/9/7	109/28/42	31/7/2	87/31/2
History of depression, n (%)	12 (26.7)	8 (4.5)	15 (37.5)	2 (1.7)
Discontinuance rate of treatment due to depression, n (%)	2 (4.4)	3 (1.7)	1 (2.5)	1 (0.8)
Baseline HCV-RNA	6.19 (0.89)	6.12 (0.91)	6.07 (0.83)	6.20 (1.01)
Baseline ALT (IU/L)	55.1 (57.4)	69.6 (80.2)	74.4 (58.1)	62.2 (48.8)
Baseline γ-GTP (IU/L)	48.3 (34.4)	52.6 (57.0)	72.3 (76.0)	51.9 (42.8)
Baseline Neutrophil (/μL)	2794.2 (1119.7)	2453.1 (1050.7)	2962.8 (1490.2)	2526.0 (978.2)
Baseline Hemoglobin (g/dL)	13.6 (1.5)	13.9 (1.5)	14.5 (1.5)	14.1 (1.8)
Baseline Platelet (10^4^/μL)	18.0 (5.4)	16.2 (5.2)	18.3 (6.7)	16.4 (5.9)

GWAS, genome-wide association study; N.D., not determined; IFN, interferon; PEG-IFN, pegylated interferon; SVR, sustained virological response; ALT, alanine aminotransferase; γ-GTP, γ-glutamyl transpeptidase; HCV, hepatitis C virus; IU, international units.

Data are expressed as number for categorical data or the mean (standard deviation) for non-categorical data.

At the stage of GWAS, we genotyped 224 Japanese HCV-infected patients: 45 patients with depression (Case-G) versus 179 patients without depression (Control-G) based on the criteria described in the Materials and Methods. S1 Fig shows a genome-wide view of single-point association data based on allele frequencies in the GWAS stage. No association between SNPs and IFN-induced depression reached a genome-wide level of significance (*P*<5.00×10^−8^) at the GWAS stage. PCA plots and QQ plots for each sample are shown in S2 Fig. The genomic inflation factor (λ) estimate was 1.013. The QC call rate for GWAS samples was divided into case and control groups in S1 Table. GWAS samples achieved the criteria for DNA samples by call rate, heterozygosity check, and IBD check.

We selected 31 SNPs with *P*<10^−4^ at GWAS, and added 11 SNPs that are located around the candidate gene regions identified by the GWAS stage and are non-synonymous or related to diseases in previous reports (S2 and S3 Tables). Consequently, we performed replication analysis focusing on a total of 42 SNPs.

In the subsequent replication analysis, we performed the genotyping of 42 candidate SNPs using the DigiTag2 assay in an independent set of 160 subjects: 40 patients with depression (Case-R) versus 120 patients without depression (Control-R). The results in the replication stage combined with GWAS are shown in S2 Table.

Consequently, the SNP rs1863918 located on chromosome 5 showed a strong association with IFN-induced depression in the combined results of the GWAS and replication stage in the allele frequency model (odds ratio = 2.55, 95% confidence interval = 1.80–3.61, *P* = 7.89×10^−8^) (Table 2). Although slightly lower than the genome-wide significance level, the combined results reached the Bonferroni criterion *P*<9.07×10^−8^ (0.05/551,176).

**Table 2:** SNP associated with IFN-induced depression.

dbSNP rsID	Nearest gene	Risk allele	Allele (1/2)	Stage	Case			Control			OR ^a^ (95% CI)	*P*-value ^b^
					11	12	22	11	12	22		
rs1863918	*ZNF354C*-	T	T/G	GWAS	12	25	8	15	79	85	2.73	2.05×10^−5^
	*ADAMTS2*				(26.7)	(55.6)	(17.8)	(8.4)	(43.6)	(48.0)	(1.70–4.38)	
				Replication	9	23	8	15	44	61	2.36	9.81×10^−4^
					(22.5)	(57.5)	(20.0)	(12.5)	(36.7)	(50.8)	(1.41–3.95)	
				Combined ^c^	21	48	16	30	123	146	2.55	7.89×10^−8^
					(24.7)	(56.5)	(18.8)	(10.0)	(40.8)	(49.2)	(1.80–3.61)	

SNP, single nucleotide polymorphism; GWAS, genome-wide association study; OR, odds ratio; CI, confidence interval; IFN, interferon. Data of allele distribution represent number (%). Data of subjects whose genotypes were not determined were excluded.

^a^ Odds ratio for the allele frequency model.

^b^ *P*-value by the chi-square test for the allele frequency model.

^c^ Allele distributions in GWAS and Replication were combined.

**Fig 2:**
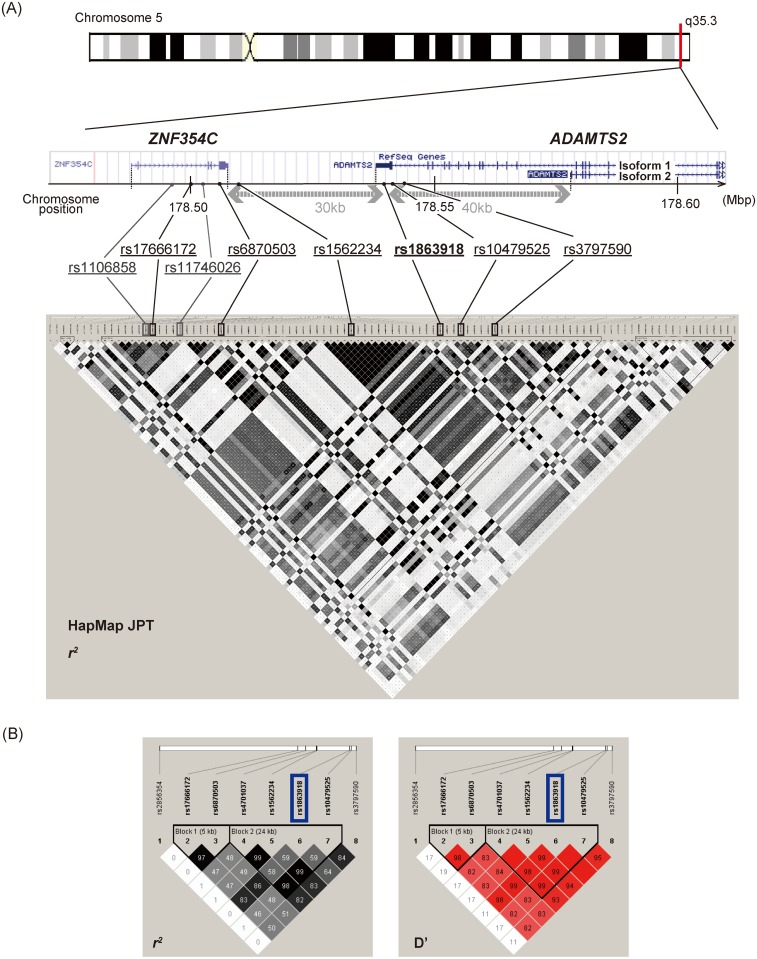
Pairwise linkage disequilibrium (*r*^2^) diagrams around the *ZNF354c-ADAMTS2* locus. (A) Position on chromosome and pairwise linkage disequilibrium (*r*^2^) diagrams in the HapMap JPT around the *ZNF354c-ADAMTS2* locus. *ADAMTS2* consists of two isoforms; a long form (isoform 1) generally identified as a conventional form and a short form (isoform 2). The SNP rs1863918 lies in the 3ʹ-UTR of *ADAMTS2* isoform 1, around 30 kb downstream from *ZNF354C*, and about 40 kb distant from the *ADAMTS2* isoform 2. (B) Estimates of pairwise *r*^2^ for 8 SNPs on chromosome 5 in the combined data set of the GWAS and replication samples. The SNPs rs17666172 and rs6870503 were in strong LD with rs1863918 (*r*^2^ = 0.83 for rs17666172; *r*^2^ = 0.86 for rs6870503).

The SNP rs1863918 lies in the 3ʹ-UTR (three prime untranslated region) of the ADAM metallopeptidase with thrombospondin type 1 motif 2 (*ADAMTS2*) gene, ~30kb downstream from the *ZNF354C* gene (Fig 2A).

### Association of SNPs located in *ZNF354C-ADAMTS2* with depression

To identify further variants, we performed SNP imputation for all genetic regions including SNPs with *P*<10^−4^ at GWAS as described in the Materials and Methods. Regional Manhattan plots around SNPs with *P*<10^−4^ obtained by SNP imputation are shown in S3 Fig. Three SNPs, rs1562234, rs10479525 and rs3797590, showed a stronger association with IFN-induced depression (*P* = 6.99×10^−6^ for rs1562234 and rs10479525, *P* = 1.80×10^−5^ for rs3797590) compared with rs1863918 (*P* = 2.05×10^−5^) (S3 Fig). However, based on genotyping data of all samples by the DigiTag2 assay, rs1863918 showed the strongest association (*P* = 7.89×10^−8^) (Table 2), compared with the other SNPs (*P* = 8.84×10^−6^ for rs1562234 and rs10479525, *P* = 1.75×10^−5^ for rs3797590 in the allele frequency model) (Table 3).

**Table 3:** Association of SNPs located in *ZNF354C-ADAMTS2* with IFN-induced depression.

dbSNP rsID	Nearest gene	Risk allele	Allele (1/2)	Case-G+R ^a^ (n = 85)			Control-G+R ^b^ (n = 299)			OR ^c^ (95% CI)	*P*-value ^d^
				11	12	22	11	12	22		
rs1562234	*ZNF354C-ADAMTS2*	G	G/A	34	39	12	58	145	96	2.19	8.84×10^−6^
				(40.0)	(45.9)	(14.1)	(19.4)	(48.5)	(32.1)	(1.54–3.11)	
rs10479525	*ADAMTS2*	T	T/G	34	39	12	59	143	97	2.19	8.84×10^−6^
				(40.0)	(45.9)	(14.1)	(19.7)	(47.8)	(32.4)	(1.54–3.11)	
rs3797590	*ADAMTS2*	A	A/G	33	40	12	50	149	100	2.32	1.75×10^−6^
				(38.8)	(47.1)	(14.1)	(16.7)	(49.8)	(33.4)	(1.64–3.30)	
rs17666172	*ZNF354C*	C	C/T	23	48	14	39	126	134	2.39	5.71×10^−7^
				(27.1)	(56.5)	(16.5)	(13.0)	(42.1)	(44.8)	(1.69–3.38)	
rs6870503	*ZNF354C*	T	T/C	21	50	14	38	129	132	2.26	2.78×10^−6^
				(24.7)	(58.5)	(16.5)	(12.7)	(43.1)	(44.1)	(1.60–3.20)	

SNP, single nucleotide polymorphism; IFN, interferon; OR, odds ratio; CI, confidence interval. Data of allele distribution represent number (%). Data of subjects whose genotypes were not determined were excluded.

^a^ Case-G+R: Case-G plus Case-R.

^b^ Control-G+R: Control-G plus Control-R.

^c^ Odds ratio for the allele frequency model.

^d^ *P*-value by the chi-square test for the allele frequency model.

Previous reports indicated that the ZNF354C protein was highly expressed in the brain but that its expression level in peripheral tissues was very low in adults [24, 25]. In contrast, the expression levels of the ADAMTS2 protein were reported to be very low in the brain [26]. Therefore, we analyzed the association with IFN-induced depression focusing on *ZNF354C*. To identify the SNPs located in *ZNF354C*, which might be in strong linkage disequilibrium (LD) with rs1863918, we examined the pairwise LD (*r*^2^) in the HapMap-JPT (Japanese in Tokyo), using Haploview version 4.2 (http://www.broadinstitute.org/haploview/haploview), a software package that provides computation of LD statistics and population haplotype patterns from primary genotype data. No missense SNPs in LD (*r*^2^>0.5) with rs1863918 were identified in the coding region of *ZNF354C*.

As shown in Fig 2A, four SNPs located in *ZNF354C*, rs1106858, rs17666172, rs11746026 and rs6870503, were in strong LD (*r*^2^ = 0.91) with rs1863918, and these SNPs were in complete LD (*r*^2^ = 1) with each other in the HapMap-JPT. The SNP rs6870503 is located in the 3ʹ-UTR of *ZNF354C* and the other SNPs are in the intron. Thus, we selected rs17666172 and rs6870503 to be genotyped by ABI TaqMan SNP assays because these SNPs were not included in the Affymetrix Genome-Wide Human SNP Array 6.0. These SNPs also showed strong associations in the combined data set of the GWAS and replication samples in the allele frequency model (*P* = 5.71×10^−7^ for 17666172, *P* = 2.78×10^−6^ for rs6870503) (Table 3). Next, we examined the pairwise LD (*r*^2^) for 8 SNPs on chromosome 5 in a total of 384 subjects of the GWAS and replication stages, and these SNPs were in strong LD with rs1863918 (*r*^2^ = 0.83 for rs17666172; *r*^2^ = 0.86 for rs6870503) (Fig 2B). These results suggest that rs1863918 was significantly associated with IFN-induced depression at GWAS and was in strong LD with SNPs located in *ZNF354C*. Therefore, the *ZNF354C* gene may have an effect on IFN-induced depression.

### Predictive factors for IFN-induced depression

The following analyses were performed for the 6 SNPs described above (rs17666172, rs6870503, rs1562234, rs1863918, rs10479525, and rs3797590) in 384 combined subjects of Case-G+R and Control-G+R. Age, a history of depression, and the genotypes of the 6 SNPs at baseline were significantly associated with IFN-induced depression by univariate analysis (Table 4). The proportion of patients who received anti-depression agents, including anxiolytic (e.g. etizolam) and antipsychotic (e.g. sulpiride, aripiprazole) drugs, during IFN-based therapy is significantly higher in the case group (n = 43, 50.6%) than in the control group (n = 8, 2.7%) (*P* = 1.25×10^−29^, by chi square test). To evaluate the association between a history of depression and the rs1863918 genotypes, we stratified the study subjects into two groups according to the rs1863918 genotypes, TT/TG group (n = 221) and GG group (n = 163), and examined the proportion of patients with or without a history of depression in each group. The proportion of patients with a history of depression was significantly higher in patients with rs1863918 TT/TG (n = 29, 13.1%) than in those with GG (n = 8, 4.9%) (*P* = 0.007, by chi-square test) (S4 Fig).

**Table 4:** Univariate analysis of pretreatment factors associated with IFN-induced depression.

	Case-G+R ^a^	Control-G+R ^b^	*P*-value ^c^
	(n = 85)	(n = 299)	
Age	53.0 (11.5)	57.6 (11.2)	3.37×10^−4^
Gender (Male/Female)	41/44	153/146	N.S.
Genotype (1/2/N.D.)	52/33/0	206/91/2	N.S.
Liver fibrosis (F0-2/F3-4/N.D.)	39/8/38	141/51/107	N.S.
Type of IFN (Peg-IFN-α2a/Peg-IFN-α2b/IFN-β)	15/60/10	67/214/18	N.S.
Period for administration of IFN (Weeks)	34.0 (17.5)	34.9 (16.6)	N.S.
Effect of treatment (rate of SVR, %)	78.8	69.2	N.S.
*IL28B*, rs8099917 (TT/TG+GG/N.D.)	60/16/9	196/59/44	N.S.
rs17666172 (TT/CT+CC)	14/71	134/165	2.16×10^−6^
rs6870503 (CC/TC+TT)	14/71	132/167	3.51×10^−6^
rs1562234 (TT/CT+CC)	12/73	96/203	0.001
rs1863918 (GG/TG+TT)	16/69	147/152	5.92×10^−7^
rs10479525 (GG/TG+TT)	12/73	97/202	9.45×10^−4^
rs3797590 (GG/AG+AA)	12/73	100/199	5.42×10^−4^
History of depression, n (%)	27 (31.8)	10 (3.3)	1.29×10^−14^
Discontinuance rate of treatment due to depression, n (%)	3 (3.5)	4 (1.3)	N.S.
Baseline HCV-RNA	6.13 (0.86)	6.15 (0.95)	N.S.
Baseline ALT (IU/L)	64.2 (58.2)	66.6 (69.4)	N.S.
Baseline γ-GTP (IU/L)	60.0 (59.4)	52.3 (51.5)	N.S.
Baseline Neutrophil (/μL)	2865.8 (1283.1)	2479.2 (1024.0)	N.S.
Baseline Hemoglobin (g/dL)	14.0 (1.6)	14.0 (1.7)	N.S.
Baseline Platelet (10^4^/μL)	18.2 (6.0)	16.3 (5.5)	0.009

N.D., not determined; IFN, interferon; PEG-IFN, pegylated interferon; N.S., not significant; SVR, sustained virological response; ALT, alanine aminotransferase; γ-GTP, γ-glutamyl transpeptidase; HCV, hepatitis C virus; IU, international units. Data are expressed as number for categorical data or the mean (standard deviation) for non-categorical data.

^a^ Case-G+R: Case-G plus Case-R.

^b^ Control-G+R: Control-G plus Control-R.

^c^ Categorical variables were compared between groups by the chi square test and non-categorical variables by the Mann-Whitney *U*-test.

Furthermore, to examine the pretreatment predictive factors for IFN-induced depression, we used logistic regression models that included the significant factors by univariate analysis and existing knowledge of risk factors for IFN-induced depression, such as age, gender, type of IFN, period for administration of IFN and a history of depression, as well as the genotypes of 6 SNPs with *P*<0.05 in univariate analysis (rs17666172, rs6870503, rs1562234, rs1863918, rs10479525, and rs3797590). The results showed that the rs1863918 T allele was an independent predictive factor for IFN-induced depression (*P* = 3.22×10^−5^) in addition to a history of depression (*P* = 1.27×10^−8^) and younger age (*P* = 0.015) (Table 5).

**Table 5:** Logistic regression analysis of treatment factors associated with IFN-induced depression.

	OR	95% CI	*P*-value
History of depression	10.69	(4.73–24.18)	1.27×10^−8^
rs1863918, T allele	3.85	(2.04–7.28)	3.22×10^−5^
Age, years	0.97	(0.95–0.99)	0.015

IFN, interferon; CI, confidence interval; OR, odds ratio.

### Expression of the ZNF354c protein in the brain

To examine the expression level of the candidate gene in the brain, we performed real-time PCR, western blotting and immunofluorescence analysis in mice, as described in the Materials and Methods.

**Fig 3:**
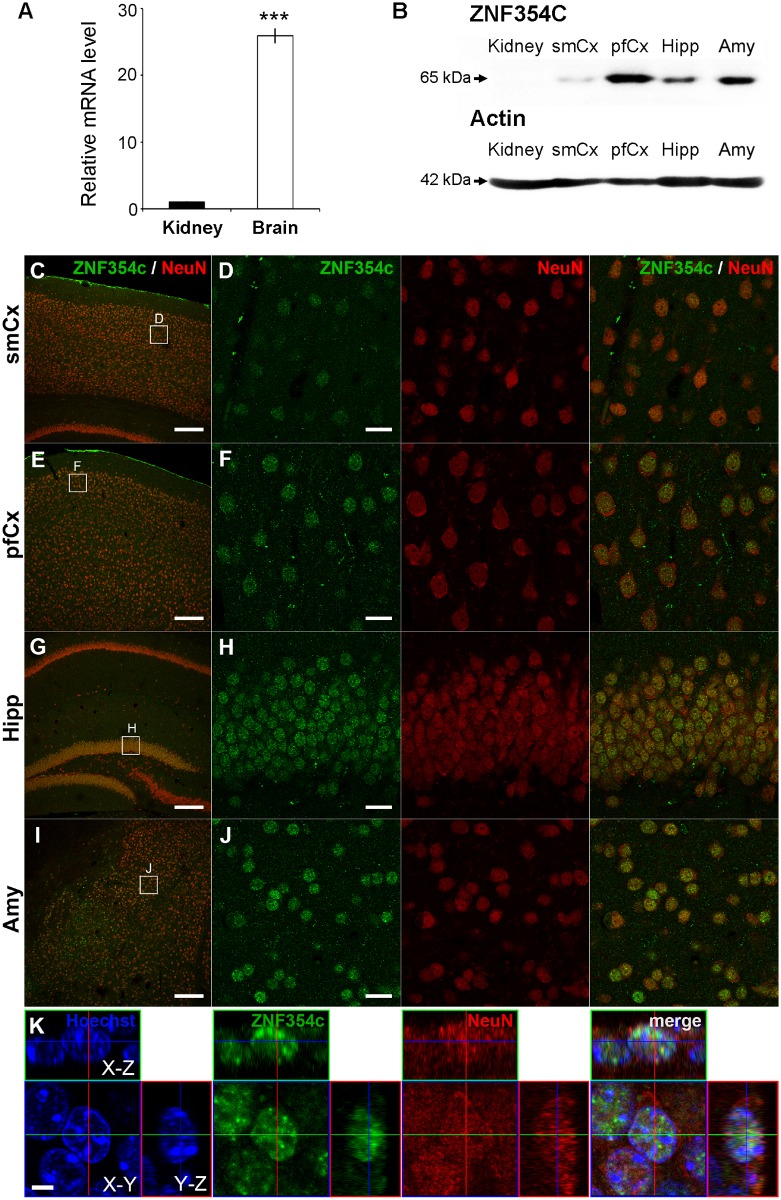
Expression of ZNF354C. (A) Relative *ZNF354C* mRNA levels in the kidney and brain in mice, quantified by real-time PCR. *ZNF354C* mRNA levels in the brain were significantly higher than in the kidney (error bars: ± SEM, *P*<0.001). (B) Western blotting of the ZNF354C protein in the kidney and brain regions [sensorimotor cortex (smCx), prefrontal cortex (pfCx), hippocampus (Hipp) and amygdala (Amy)] in mice. ZNF354C protein (65 kDa) was highly expressed in the pfCx, Hipp and Amy, the brain regions implicated in the pathology of psychiatric symptoms (top). Actin (42 kDa) was used as a control (bottom). (C)–(J) Immunofluorescence of ZNF354C (green) and NeuN (red), a marker for mature neurons, in the smCx (C, D), pfCx (E, F), Hipp (G, H) and Amy (I, J). D, F, H and J are higher magnification images of the boxed areas in C, E, G and I, respectively. (K) Nuclear localization of ZNF354C. Confocal images (X-Y, X-Z and Y-Z planes) of a neuron in the dentate gyrus of the hippocampus show that ZNF354C (green) in a NeuN-positive neuron (red) is localized to its nucleus (Hoechst, blue). Scale bars, C, E, G, I, 200 μm; D, F, H, J, 20 μm; K, 5 μm.

We compared relative mRNA levels of *ZNF354C* and *ADAMTS2* in the brain, quantified by real-time PCR. *ZNF354C* mRNA was highly expressed in the brain, whereas *ADAMTS2* mRNA expression levels were very low. Thus, we analyzed the expression levels of *ZNF354C* alone, and the results indicated that *ZNF354C* mRNA levels in the brain were significantly higher than in the kidney (*P*<0.001) (Fig 3A).

We also performed western blotting and immunofluorescence analysis of the ZNF354C protein in the kidney and various regions of the brain [sensorimotor cortex (smCx), prefrontal cortex (pfCx), hippocampus (Hipp) and amygdala (Amy)]. Some previous reports stated that *ZNF354C* mRNA was not detected in liver of mice at any of the developmental stages, while *ZNF354C* mRNA expression was detectable in kidney of mice, with expression markedly-decreased postnatally [24, 25, 27]. Therefore, we selected the kidney rather than the liver for comparison to examine the expression of ZNF354C in the brain regions. Western blotting showed that the ZNF354C protein was highly expressed in the pfCx, Hipp and Amy (Fig 3B). Interestingly, these brain regions have been implicated in the pathology of psychiatric symptoms. Immunofluorescence analysis also showed that ZNF354C was mainly localized to the nuclei in neurons of all the regions examined (Fig 3C–3J). Confocal images of a neuron in the dentate gyrus of the hippocampus confirmed that ZNF354c in a NeuN-positive neuron was localized to its nucleus (Fig 3K).

Furthermore, to investigate whether *ZNF354C* mRNA was induced in the brain by the administration of IFN-α, we quantified relative *ZNF354C* mRNA levels in the brain in mice treated with phosphate buffered saline (PBS) or IFN-α for 4 weeks (n = 6 mice per group) by real-time PCR. *ZNF354C* mRNA levels in the brain of IFN-α-treated mice were slightly higher than those of the PBS-treated controls (control: 1.01±0.08, IFN-α: 1.37±0.07, *P* = 0.00892, n = 6 mice per group). Thus, these results indicate that *ZNF354C* mRNA expression in the brain might be increased by the long-term administration of IFN-α.

## Discussion

Although it is known that IFN administration is likely to induce depression in patients with a history of depression, no reliable information regarding the genetic factors involved in the onset of IFN-induced depression has been reported [28]. To the best of our knowledge, no studies have demonstrated a SNP associated with IFN-induced depression at the genome-wide significance level. In this study, rs1863918 was identified by GWAS as a SNP associated with IFN-induced depression; patients with the rs1863918 T allele have a significantly increased risk for IFN-induced depression. The association nearly reached the genome-wide significance level. Furthermore, patients with T allele tended to be more likely to have a history of depression. However, this finding needs to be verified with larger sample numbers because the numbers of patients with a history of depression are very small. The allele frequency of rs1863918 in the public database showed that 64% of Japanese individuals have G allele (G/T, 0.64/0.36) according to HapMap-JPT. There is no evidence that rs1863918 is associated with depression in the PGC (Psychiatric Genomics Consortium; http://www.med.unc.edu/pgc/) public GWAS data. The SNP rs1863918 is located at the 3ʹ-UTR of *ADAMTS2* on chromosome 5. In addition to *ADAMTS2*, the *ZNF354C* gene is also adjacent to the SNP. Interestingly, SNPs that showed LD with the rs1863918 (*r*^*2*^ = 0.83–0.91) cluster at the 3ʹ-UTR (rs6870503) and intron (rs1106858, rs17666172, and rs11746026) of *ZNF354C*. Our study suggests that *ZNF354C* is highly expressed in the hippocampus and might be implicated in the pathology of depression.

*ADAMTS2* encodes a peptidase (collagen synthase) and extracellular excreted protein that has been reported to be responsible for Ehlers-Danlos syndrome (recessive heredity) [29] and accelerate liver fibrosis [30]; however, its expression is low in the brain [26]. *ZNF354C* encodes a transcriptional repressor that is involved in ischemia, bone formation, and differentiation [27]. Although it was reported that it is highly expressed in the brain [24, 25], published information on the function of this protein is limited.

*ADAMTS2* consists of two isoforms, a conventional long form (isoform 1) and a short form (isoform 2). The SNP rs1863918 is located on the 3ʹ-UTR of isoform 1 of *ADAMTS2* and is about 30 kb downstream of *ZNF354C*, and about 40 kb from isoform 2 (Fig 2A). To investigate the expression of *ADAMTS2* and *ZNF354C* in the brain, RNA-seq read mapping was performed with Human Protein Atlas version 13 (http://www.proteinatlas.org), and their expressions in the brain and liver were compared (S5 Fig). The overall expression level of *ZNF354C* was higher in the brain than in the liver (about 2–7-fold by Fragments Per Kilobase of exon per Million mapped fragments [FPKM]) with a high peak at the end of the 3ʹ-UTR. However, *ADAMTS2* was highly expressed at the last exon/3ʹ-UTR of isoform 1 in the liver, but its expression level was relatively lower in the brain than in the liver (about 4–10-fold by FPKM) and markedly low at the 3ʹ-UTR. Because isoform 1 of *ADAMTS2* is expressed at a low level in the brain, isoform 2 might be the predominant brain isoform, suggesting the nearest gene of rs1863918 might be *ZNF354C*. *ZNF354C* was highly expressed in the brain with a peak at the 3ʹ-UTR.

Furthermore, to investigate the expression of the candidate genes *ZNF354C* and *ADAMTS2* in the brain and their induction by IFN-α *in vivo*, mRNA was quantified by real-time PCR and its function was analyzed by western blotting and immunofluorescence in mice. We found that *ZNF354C* was highly expressed in the brain, especially in the hippocampus (Fig 3). It has been recently reported that IFN-α-induced depression may be caused by decreased neurogenesis in the hippocampus [31]. Because the expression level of *ZNF354C* was high in the hippocampus, it is suggested *ZNF354C* might be involved in the onset of depression. The expression level of *ZNF354C* in the brain tended to be higher in mice treated with IFN-α for four weeks, suggesting IFN-α might induce *ZNF354C* expression in the brain.

Next, the transcription factor binding site (TFBS) in the rs1863918 region was estimated with HaploReg version 4 (http://www.broadinstitute.org/mammals/haploreg/haploreg.php), a tool to explore regulatory motif alterations, and Zinc finger and BTB domain containing 3 (*ZBTB3*) was identified as an altered regulatory motif. The regulatory motif sequence of *ZBTB3* is VVVMVTGCAGTGSVNNH and when rs1863918 SNP was G allele, which is a reference allele, the log-odds score was 8.7, and when rs1863918 SNP was T allele, an alternative allele, the score was elevated to 12.7 [32]. The rs1863918 G allele has no function, and the rs1863918 T allele functions as a regulatory sequence that may control the transcription of *ZNF354C* (positive feedback) via recognition of the *ZBTB3* regulatory motif that binds to the enhancer element.

ZBTB3 proteins contain a DNA binding zinc finger at the C-terminus and a transcription-repressing BTB/POZ domain at the N-terminus, and function as transcriptional repressors via the BTB/POZ domain-mediated recruitment of a variety of transcriptional co-repressors to a subset of their target gene promoter region [33]. It has been reported that *ZBTB3* is a transcription regulatory factor that either activates or suppresses transcription according to the cellular context and which is essential for the proliferation of cancer cells [34]. Furthermore, it has been suggested that *ZBTB3* is involved in the transcriptional regulation of antioxidative substances (enzymes) and is potentially influential in the reactive oxygen species (ROS) pathway of oxidative stress [34, 35]. In addition, *ZBTB3* might be an interacting protein for the glucocorticoid receptor (GR) in rat hippocampus neurons to regulate transcription in a GR-dependent manner [36, 37].

Regarding the onset mechanism for IFN-induced depression, it was recently reported that inflammatory cytokines [38] and oxidative stress induced by ROS production [35] were influential and that continuously elevated glucocorticoid concentrations induced by disrupting the negative feedback mechanism in the hypothalamic-pituitary-adrenal axis brought on neuroplasticity of the hippocampus [39]. Taken together, when rs1863918 SNP is T allele, it is assumed that ZBTB3 plays a direct role in these processes by binding to the target region or has an indirect role by upregulating ZNF354C expression in the hippocampus as the onset mechanism of depression.

This study had some limitations. First, the number of case samples was small, but it is difficult to obtain more samples because of the low disease prevalence. Therefore, we performed a replication study. Secondly, we did not correct for population structures in the GWAS samples using logistic regression with ancestry covariates and did not utilize meta-analysis to combine the two cohorts. However, to avoid bias induced by population stratification, we obtained study samples from Japanese subjects reported to be a relatively homogenous population [40–42]. Thirdly, the association with the SNP rs1863918 did not reach a genome-wide significance level; however, it reached the Bonferroni criterion *P*<9.07×10^−8^ (0.05/551,176), and we suggest that the candidate gene *ZNF354C* might be a disease-related gene, considering the results of animal experiments as well as GWAS. Finally, the assumptions about the role of ZBTB3 are based on database analyses and the mechanisms cannot be demonstrated by functional analyses at present. Further studies incorporating functional analyses *in vitro* are needed to elucidate these mechanisms.

In conclusion, this study indicates that IFN-induced depression was significantly more likely to occur in patients with rs1863918 T allele and that high *ZNF354C* expression levels in the hippocampus might be involved in the onset of depression. The genetic factor that influences IFN-induced depression might also be related to depression induced by other drugs and major depressive disorders, although the phenotype of IFN-induced depression is discriminated from endogenous depression. Therefore, these results may help to elucidate the onset mechanisms of drug-induced depression including IFN-induced depression.

## Supporting Information

S1 Fig Genome-wide association results of 224 Japanese HCV-infected patients with depression induced by IFN-based therapy (45 patients with depression and 179 patients without depression). (PDF)

S2 Fig Principal component analysis (PCA) plots and Quantile-quantile (QQ) plots for GWAS samples. (a) PCA plots for GWAS samples. (b) QQ plots of the observed versus the expected *P*-values for each samples.
(PDF)

S3 Fig Regional Manhattan plots around SNPs with *P*<10^−4^ obtained by SNP imputation. SNP imputation for the genetic regions including SNPs with *P*<10^−4^ at GWAS identified 3 SNPs with *P*<10^−4^ as follows: (a) rs1863918, (b) rs3797590 and (C) rs4904887.
(PDF)

S4 Fig History of depression according to rs1863918 genotypes. (PDF)

S5 Fig The RNA seq read mapping of *ZNF354C* and *ADAMTS2* obtained by the Human Protein Atlas ver13. (PDF)

S1 Table Quality control call rate for genome-wide association study samples divided into cases and controls. (DOCX)

S2 Table Results of replication analysis for 42 SNPs. (DOCX)

S3 Table Further details of the 11 SNPs used for replication analysis. (DOCX)
