# Supplementary material for: Genome-Wide Association Study Identifies ZNF354C Variants Associated with Depression from Interferon-Based Therapy for Chronic Hepatitis C
Source: PLoS One. 2016 Oct 10;11(10):e0164418. doi: 10.1371/journal.pone.0164418 (PMC5056723; doi:10.1371/journal.pone.0164418)
Supplement: S1 Fig — (PDF) [file pone.0164418.s001.pdf]

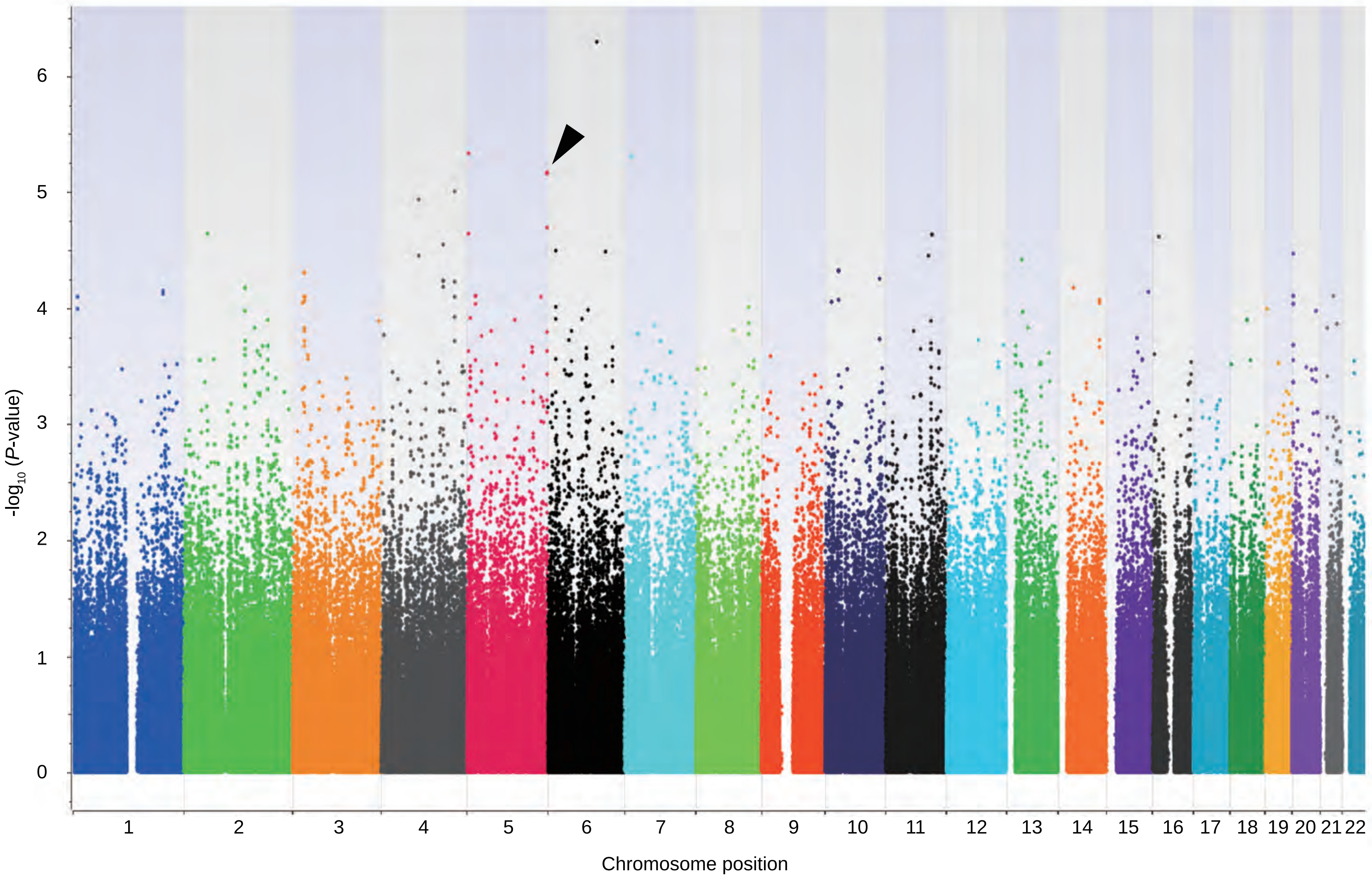

S1 Fig. Genome-wide association results of 224 Japanese HCV-infected patients with depression induced by IFN-based therapy (45 patients with depression and 179 patients without depression).

P-values were calculated using the chi-square test for allele frequencies. Dot with arrow on chromosome 5 indicates the rs1863918 SNP (P=2.05×10^{-5}) that is strongly associated with IFN-induced depression.
