# Supplementary material for: Genome-Wide Association Study Identifies ZNF354C Variants Associated with Depression from Interferon-Based Therapy for Chronic Hepatitis C
Source: PLoS One. 2016 Oct 10;11(10):e0164418. doi: 10.1371/journal.pone.0164418 (PMC5056723; doi:10.1371/journal.pone.0164418)
Supplement: S2 Fig — (a) PCA plots for GWAS samples. (b) QQ plots of the observed versus the expected P-values for each samples. (PDF) [file pone.0164418.s002.pdf]

(a)

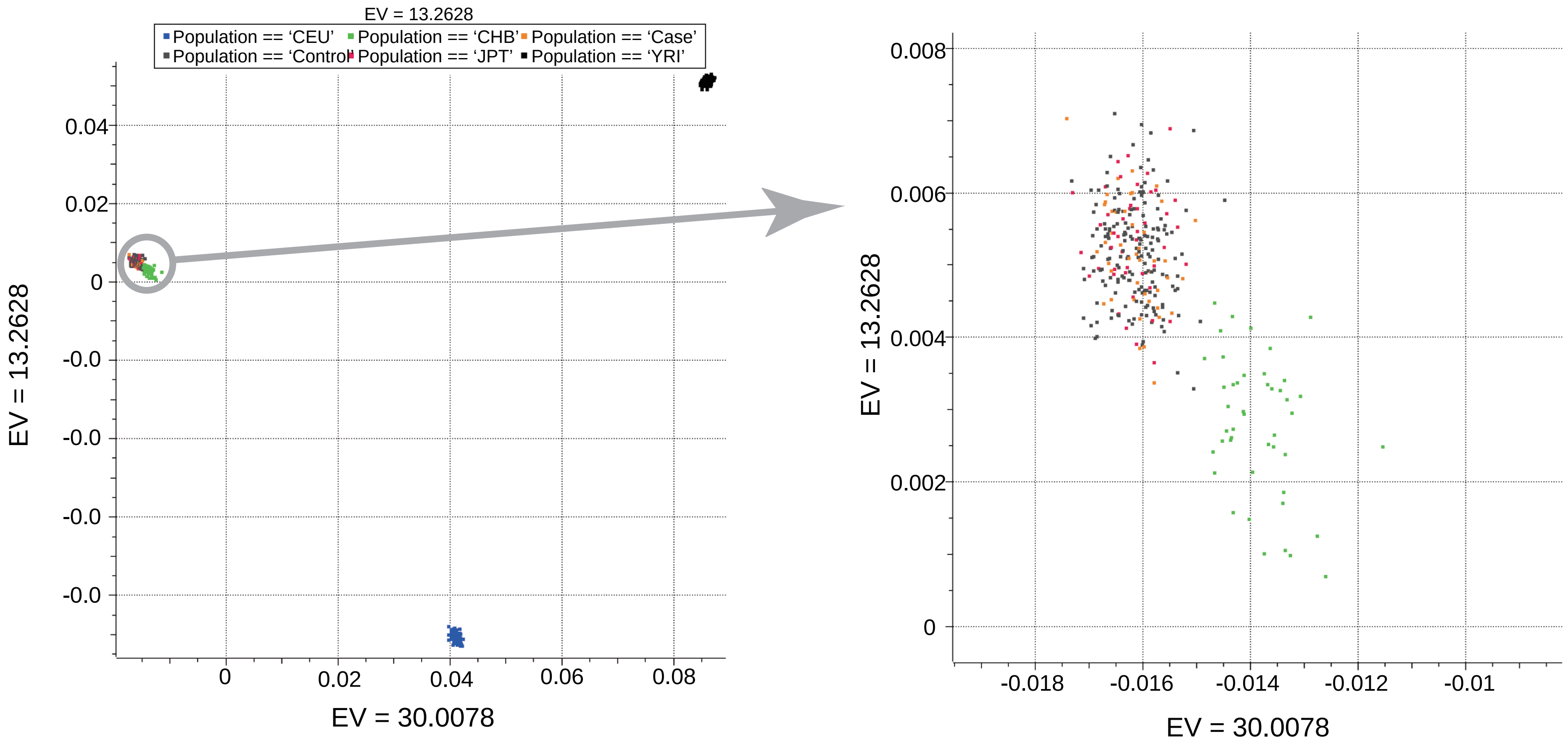

S2 Fig. Principal component analysis (PCA) plots and Quantile-quantile (QQ) plots for GWAS samples.
(a) PCA plots for GWAS samples. Orange dots indicate case samples and grey dots indicate control samples. Case and control samples are distributed in accordance with the population of Japanese in Tokyo (JPT). Population outliers were not identified based on the PCA.

CEU, Caucasians living in Utah of northern and western European ancestry; CHB, Han Chinese from Beijing, China; JPT, Japanese in Tokyo, Japan; YRI, Yoruba in Ibadan, Nigeria.

(b)

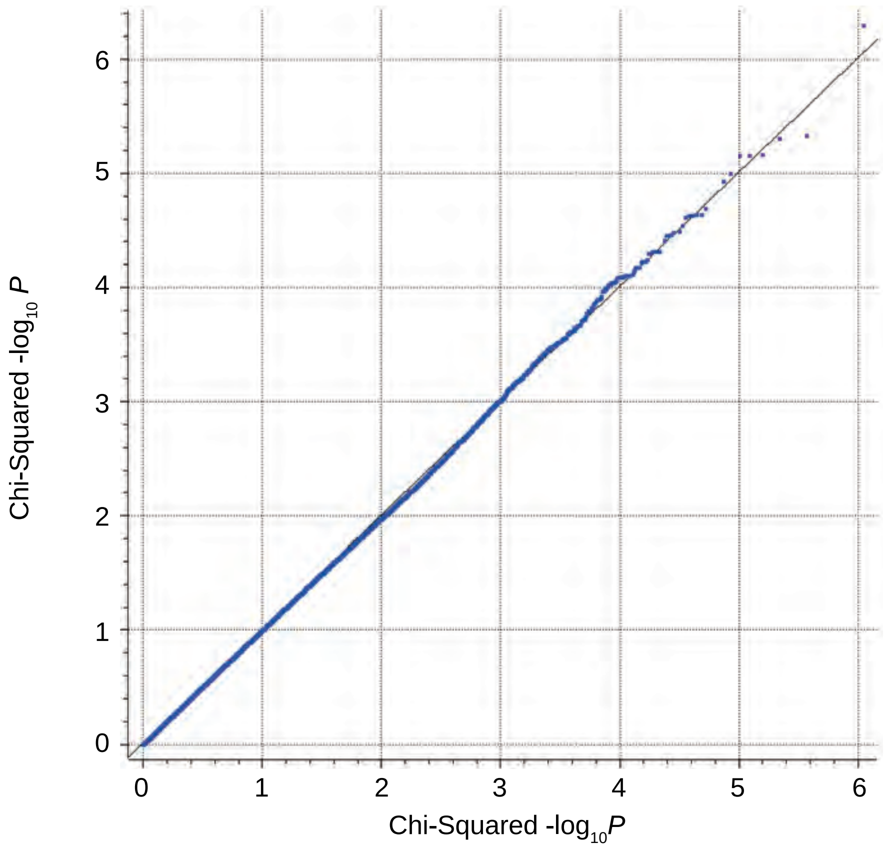

S2 Fig. Principal component analysis (PCA) plots and Quantile-quantile (QQ) plots for GWAS samples.
(b) QQ plots of the observed versus the expected *P*-values for each samples. The genomic inflation factor (λ) estimate was 1.013, indicating there was no meaningful inflation of test statistics.
