# Supplementary material for: Genome-Wide Association Study Identifies ZNF354C Variants Associated with Depression from Interferon-Based Therapy for Chronic Hepatitis C
Source: PLoS One. 2016 Oct 10;11(10):e0164418. doi: 10.1371/journal.pone.0164418 (PMC5056723; doi:10.1371/journal.pone.0164418)
Supplement: S3 Fig — SNP imputation for the genetic regions including SNPs with P<10−4 at GWAS identified 3 SNPs with P<10−4 as follows: (a) rs1863918, (b) rs3797590 and (C) rs4904887. (PDF) [file pone.0164418.s003.pdf]

(a)

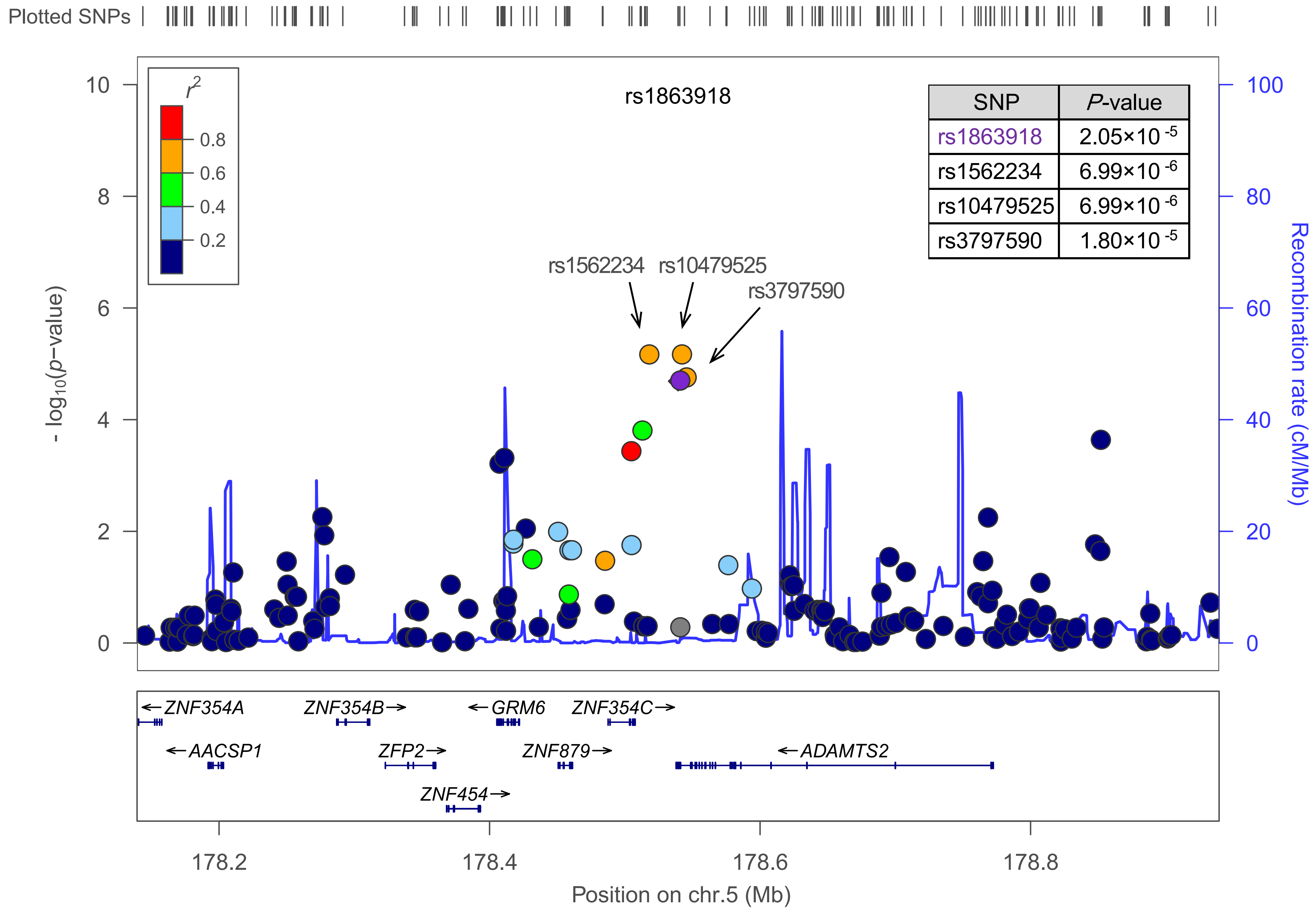

S3 Fig. Regional Manhattan plots around SNPs with $P<10^{-4}$ obtained by SNP imputation.
SNP imputation for the genetic regions including SNPs with $P<10^{-4}$ at GWAS identified 3 SNPs with $P<10^{-4}$ as follows: (a) rs1863918, (b) rs3797590 and (C) rs4904887. Purple dot indicates the reference SNP. *P*-value and odds ratio for reference SNP and SNPs with $P<10^{-4}$ are shown in the upper right.

(a) Three SNPs showed a stronger association with IFN-induced depression ($P=6.99\times10^{-6}$ for rs1562234 and rs10479525, $P=1.80\times10^{-5}$ for rs3797590) compared with rs1863918 ($P=2.05\times10^{-5}$). The SNP rs1562234 is located in the intergenic region between *ZNF354C* and *ADAMTS2*, and rs10479525 is located in the 3'-UTR of *ADAMTS2*, and rs3797590 is located in the intron of *ADAMTS2*.

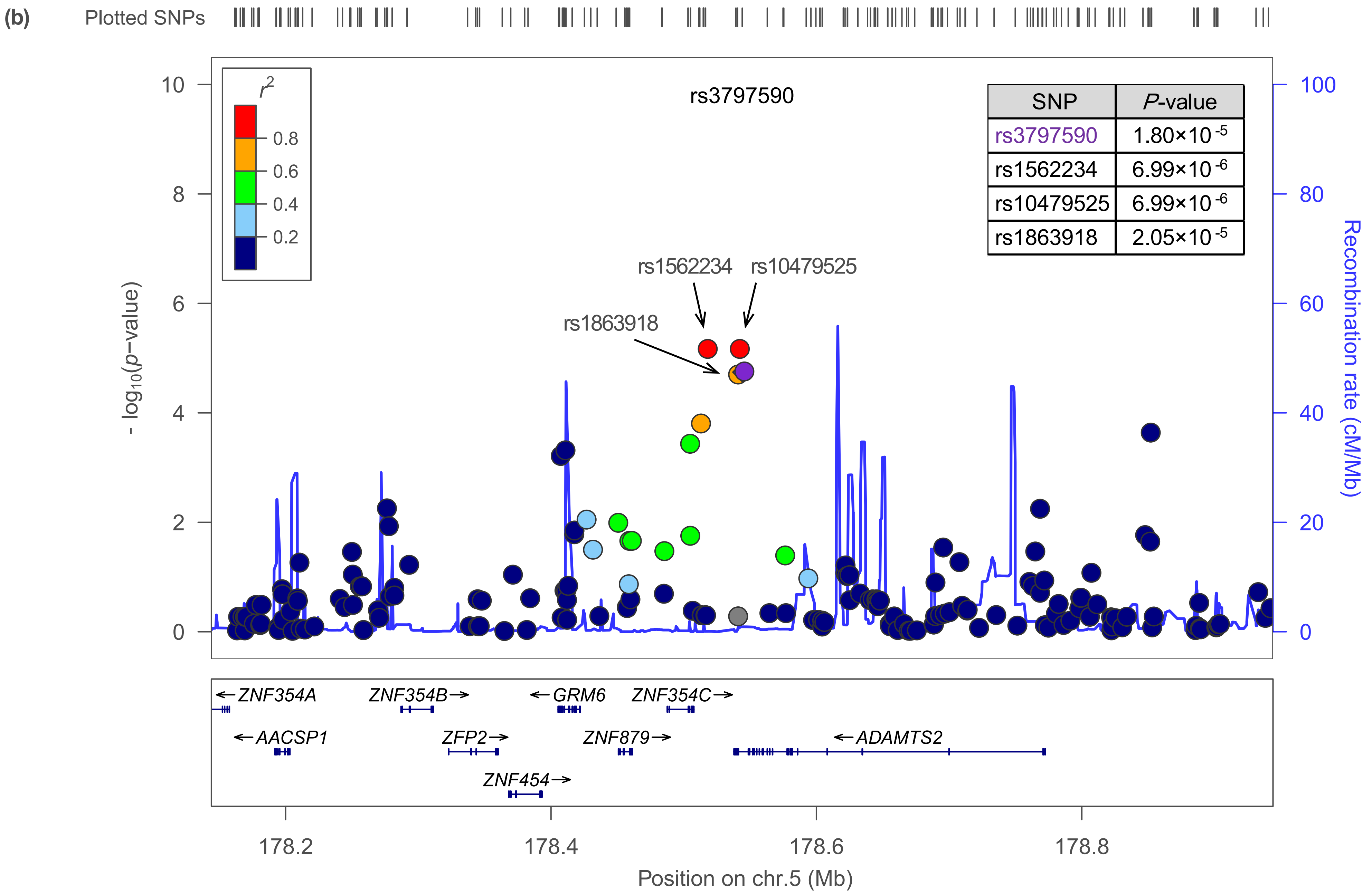

S3 Fig. Regional Manhattan plots around SNPs with *P*<10^{-4} obtained by SNP imputation.
SNP imputation for the genetic regions including SNPs with *P*<10^{-4} at GWAS identified 3 SNPs with *P*<10^{-4} as follows: (a) rs1863918, (b) rs3797590 and (C) rs4904887. Purple dot indicates the reference SNP. *P*-value and odds ratio for reference SNP and SNPs with *P*<10^{-4} are shown in the upper right.

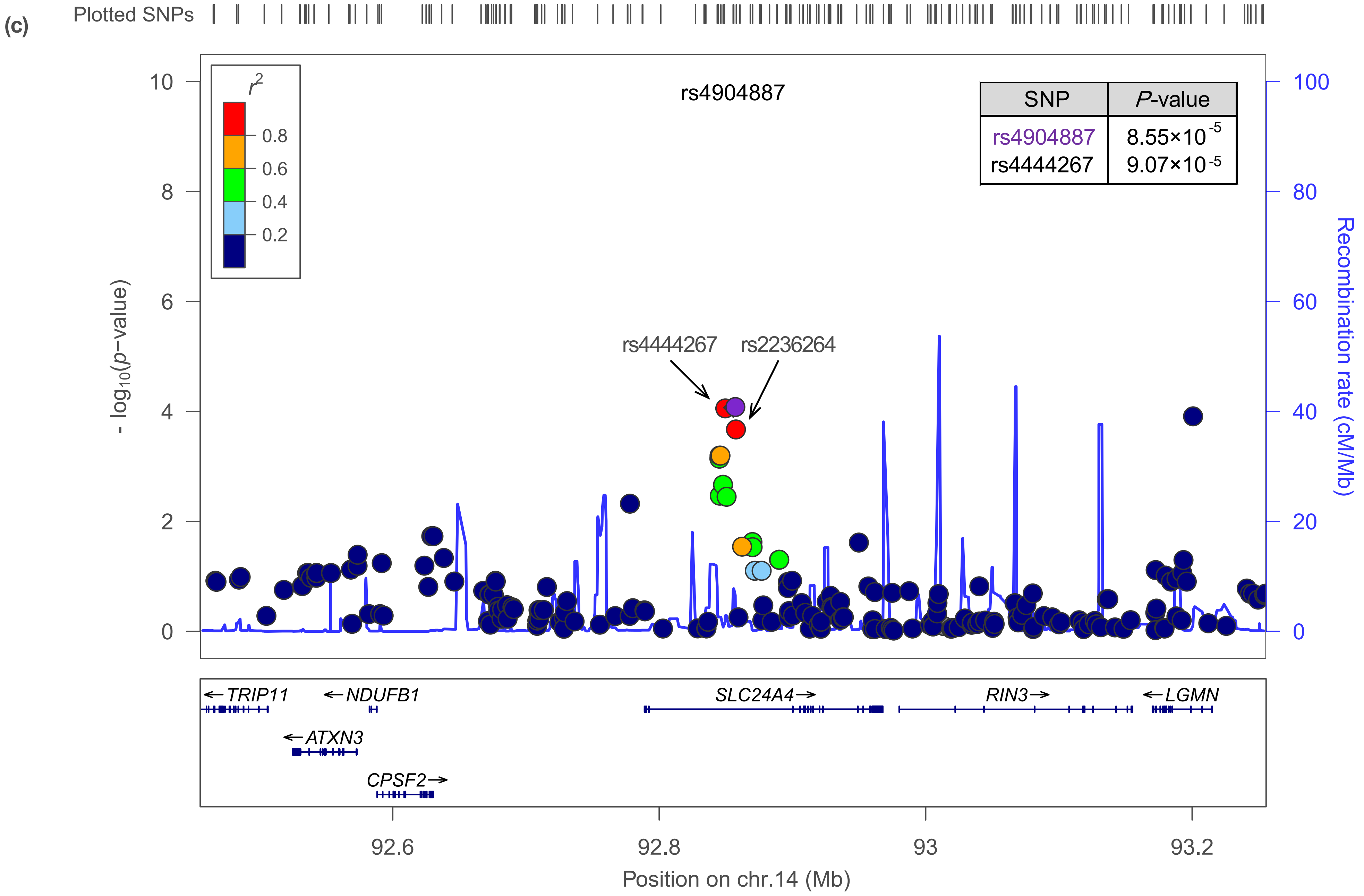

S3 Fig. Regional Manhattan plots around SNPs with P<10^{-4} obtained by SNP imputation.
SNP imputation for the genetic regions including SNPs with P<10^{-4} at GWAS identified 3 SNPs with P<10^{-4} as follows: (a) rs1863918, (b) rs3797590 and (C) rs4904887. Purple dot indicates the reference SNP. P-value and odds ratio for reference SNP and SNPs with P<10^{-4} are shown in the upper right.
