# Supplementary material for: Genome-Wide Association Study Identifies ZNF354C Variants Associated with Depression from Interferon-Based Therapy for Chronic Hepatitis C
Source: PLoS One. 2016 Oct 10;11(10):e0164418. doi: 10.1371/journal.pone.0164418 (PMC5056723; doi:10.1371/journal.pone.0164418)
Supplement: S4 Fig — (PDF) [file pone.0164418.s004.pdf]

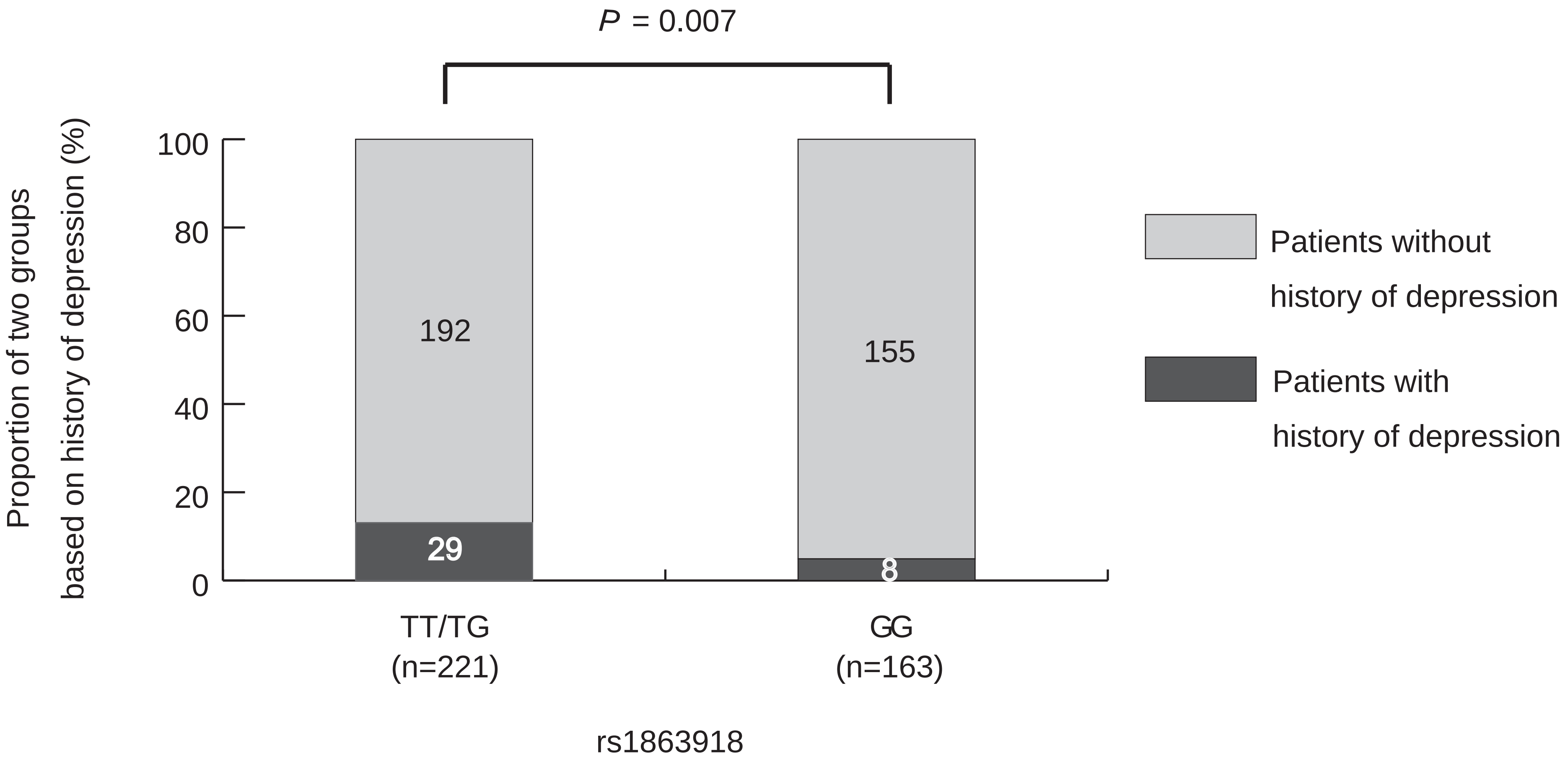

S4 Fig. History of depression according to rs1863918 genotypes.

The patients were stratified into two groups according to rs1863918 genotypes, as follows: those with TT/TG (n=221) or GG (n=163). The proportion of patients with a history of depression was significantly higher in patients with rs1863918 TT/TG than in those with GG (*P*=0.007, by chi-square test).
