# Supplementary material for: Genome-Wide Association Study Identifies ZNF354C Variants Associated with Depression from Interferon-Based Therapy for Chronic Hepatitis C
Source: PLoS One. 2016 Oct 10;11(10):e0164418. doi: 10.1371/journal.pone.0164418 (PMC5056723; doi:10.1371/journal.pone.0164418)
Supplement: S5 Fig — (PDF) [file pone.0164418.s005.pdf]

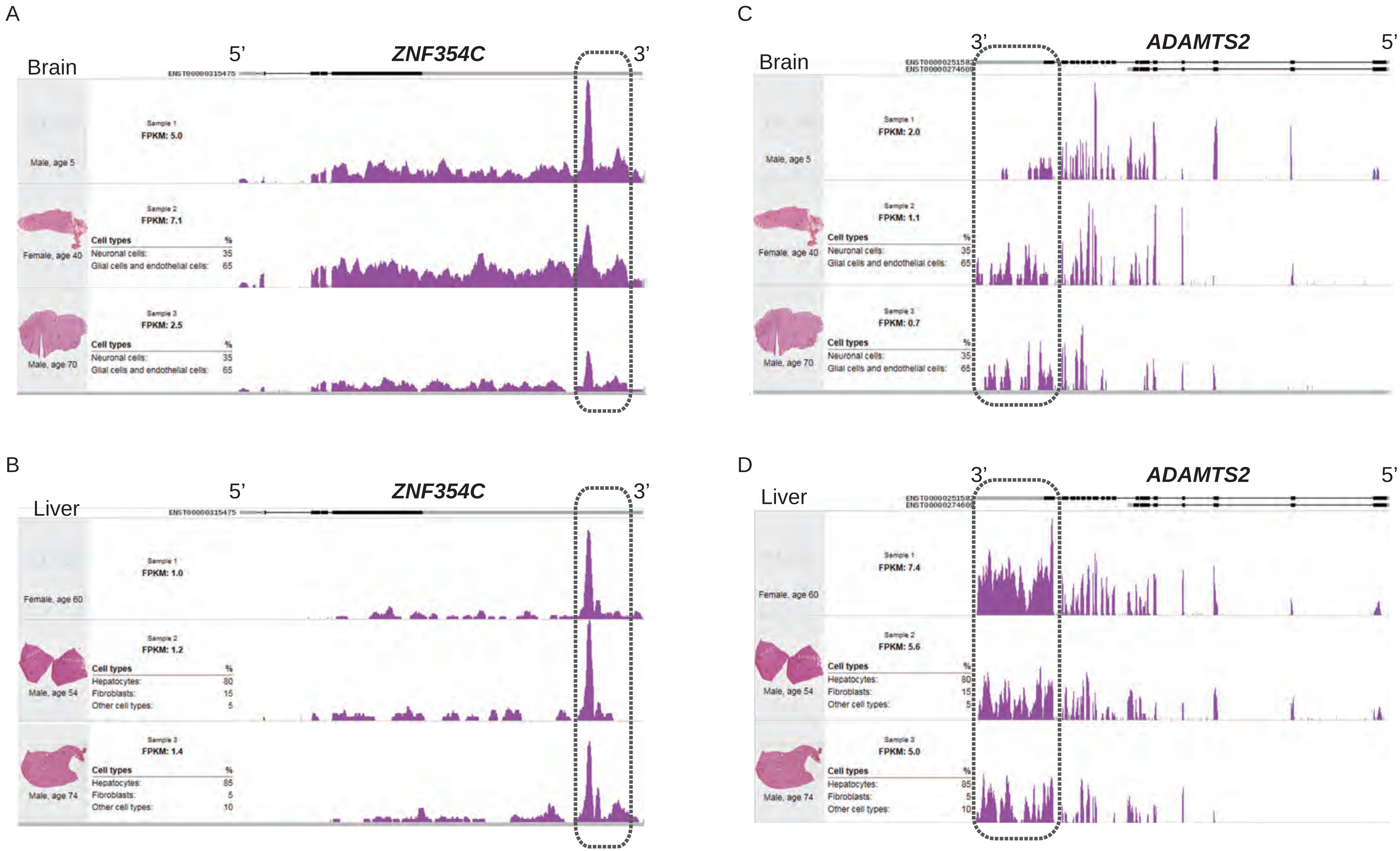

S5 Fig. The RNA seq read mapping of *ZNF354C* and *ADAMTS2* obtained by the Human Protein Atlas ver13.

The plots show the mapped read coverage along all exons and introns of the genes in each individual sample. In detail, the expression levels of *ZNF354C* in the brain (A) and liver (B) and *ADAMTS2* in the brain (C) and liver (D) are shown. The levels are normalized by total library size and scaled to fit the highest peak of all samples to the height of the plot. FPKM (Fragments Per Kilobase of exon per Million fragments mapped) values give a quantification of the gene abundance which is comparable between different genes and samples.
