# Supplementary material for: Genome-Wide Association Study Identifies ZNF354C Variants Associated with Depression from Interferon-Based Therapy for Chronic Hepatitis C
Source: PLoS One. 2016 Oct 10;11(10):e0164418. doi: 10.1371/journal.pone.0164418 (PMC5056723; doi:10.1371/journal.pone.0164418)
Supplement: S1 Table — (DOCX) [file pone.0164418.s006.docx]

**S1 Table. Quality control call rate for genome-wide association study samples divided into cases and controls.**

Case (n=45) Control (n=179)

| # | Call Rate  (Autosomes) |  | # | Call Rate  (Autosomes) |  | # | Call Rate  (Autosomes) |  | # | Call Rate  (Autosomes) |  | # | Call Rate  (Autosomes) |  | # | Call Rate  (Autosomes) |  | # | Call Rate  (Autosomes) |  | # | Call Rate  (Autosomes) |
| --- | --- | --- | --- | --- | --- | --- | --- | --- | --- | --- | --- | --- | --- | --- | --- | --- | --- | --- | --- | --- | --- | --- |
| 1 | 0.9946 |  | 31 | 0.9902 |  | 1 | 0.9928 |  | 31 | 0.9956 |  | 61 | 0.9937 |  | 91 | 0.9903 |  | 121 | 0.9887 |  | 151 | 0.9890 |
| 2 | 0.9932 |  | 32 | 0.9918 |  | 2 | 0.9951 |  | 32 | 0.9953 |  | 62 | 0.9954 |  | 92 | 0.9944 |  | 122 | 0.9956 |  | 152 | 0.9949 |
| 3 | 0.9959 |  | 33 | 0.9950 |  | 3 | 0.9951 |  | 33 | 0.9965 |  | 63 | 0.9957 |  | 93 | 0.9896 |  | 123 | 0.9944 |  | 153 | 0.9832 |
| 4 | 0.9933 |  | 34 | 0.9933 |  | 4 | 0.9946 |  | 34 | 0.9898 |  | 64 | 0.9967 |  | 94 | 0.9856 |  | 124 | 0.9953 |  | 154 | 0.9959 |
| 5 | 0.9906 |  | 35 | 0.9838 |  | 5 | 0.9935 |  | 35 | 0.9941 |  | 65 | 0.9945 |  | 95 | 0.9930 |  | 125 | 0.9941 |  | 155 | 0.9959 |
| 6 | 0.9950 |  | 36 | 0.9959 |  | 6 | 0.9930 |  | 36 | 0.9897 |  | 66 | 0.9882 |  | 96 | 0.9939 |  | 126 | 0.9958 |  | 156 | 0.9915 |
| 7 | 0.9923 |  | 37 | 0.9930 |  | 7 | 0.9939 |  | 37 | 0.9917 |  | 67 | 0.9950 |  | 97 | 0.9956 |  | 127 | 0.9896 |  | 157 | 0.9952 |
| 8 | 0.9958 |  | 38 | 0.9908 |  | 8 | 0.9928 |  | 38 | 0.9905 |  | 68 | 0.9938 |  | 98 | 0.9900 |  | 128 | 0.9905 |  | 158 | 0.9890 |
| 9 | 0.9950 |  | 39 | 0.9924 |  | 9 | 0.9942 |  | 39 | 0.9858 |  | 69 | 0.9968 |  | 99 | 0.9953 |  | 129 | 0.9946 |  | 159 | 0.9934 |
| 10 | 0.9926 |  | 40 | 0.9884 |  | 10 | 0.9909 |  | 40 | 0.9880 |  | 70 | 0.9925 |  | 100 | 0.9893 |  | 130 | 0.9954 |  | 160 | 0.9956 |
| 11 | 0.9921 |  | 41 | 0.9878 |  | 11 | 0.9859 |  | 41 | 0.9908 |  | 71 | 0.9950 |  | 101 | 0.9942 |  | 131 | 0.9952 |  | 161 | 0.9951 |
| 12 | 0.9946 |  | 42 | 0.9927 |  | 12 | 0.9933 |  | 42 | 0.9946 |  | 72 | 0.9919 |  | 102 | 0.9940 |  | 132 | 0.9926 |  | 162 | 0.9912 |
| 13 | 0.9949 |  | 43 | 0.9904 |  | 13 | 0.9943 |  | 43 | 0.9897 |  | 73 | 0.9939 |  | 103 | 0.9957 |  | 133 | 0.9950 |  | 163 | 0.9962 |
| 14 | 0.9924 |  | 44 | 0.9870 |  | 14 | 0.9927 |  | 44 | 0.9915 |  | 74 | 0.9950 |  | 104 | 0.9955 |  | 134 | 0.9941 |  | 164 | 0.9961 |
| 15 | 0.9932 |  | 45 | 0.9934 |  | 15 | 0.9960 |  | 45 | 0.9901 |  | 75 | 0.9916 |  | 105 | 0.9808 |  | 135 | 0.9903 |  | 165 | 0.9852 |
| 16 | 0.9923 |  |  |  |  | 16 | 0.9927 |  | 46 | 0.9925 |  | 76 | 0.9906 |  | 106 | 0.9957 |  | 136 | 0.9921 |  | 166 | 0.9951 |
| 17 | 0.9859 |  |  |  |  | 17 | 0.9954 |  | 47 | 0.9938 |  | 77 | 0.9946 |  | 107 | 0.9945 |  | 137 | 0.9949 |  | 167 | 0.9961 |
| 18 | 0.9930 |  |  |  |  | 18 | 0.9957 |  | 48 | 0.9910 |  | 78 | 0.9923 |  | 108 | 0.9937 |  | 138 | 0.9919 |  | 168 | 0.9926 |
| 19 | 0.9919 |  |  |  |  | 19 | 0.9927 |  | 49 | 0.9895 |  | 79 | 0.9920 |  | 109 | 0.9945 |  | 139 | 0.9936 |  | 169 | 0.9887 |
| 20 | 0.9917 |  |  |  |  | 20 | 0.9939 |  | 50 | 0.9906 |  | 80 | 0.9857 |  | 110 | 0.9950 |  | 140 | 0.9880 |  | 170 | 0.9888 |
| 21 | 0.9943 |  |  |  |  | 21 | 0.9914 |  | 51 | 0.9815 |  | 81 | 0.9917 |  | 111 | 0.9945 |  | 141 | 0.9914 |  | 171 | 0.9924 |
| 22 | 0.9933 |  |  |  |  | 22 | 0.9924 |  | 52 | 0.9923 |  | 82 | 0.9914 |  | 112 | 0.9930 |  | 142 | 0.9886 |  | 172 | 0.9939 |
| 23 | 0.9928 |  |  |  |  | 23 | 0.9934 |  | 53 | 0.9924 |  | 83 | 0.9960 |  | 113 | 0.9947 |  | 143 | 0.9904 |  | 173 | 0.9910 |
| 24 | 0.9884 |  |  |  |  | 24 | 0.9937 |  | 54 | 0.9878 |  | 84 | 0.9951 |  | 114 | 0.9964 |  | 144 | 0.9920 |  | 174 | 0.9931 |
| 25 | 0.9960 |  |  |  |  | 25 | 0.9863 |  | 55 | 0.9839 |  | 85 | 0.9960 |  | 115 | 0.9938 |  | 145 | 0.9923 |  | 175 | 0.9944 |
| 26 | 0.9932 |  |  |  |  | 26 | 0.9945 |  | 56 | 0.9872 |  | 86 | 0.9940 |  | 116 | 0.9958 |  | 146 | 0.9851 |  | 176 | 0.9917 |
| 27 | 0.9935 |  |  |  |  | 27 | 0.9949 |  | 57 | 0.9922 |  | 87 | 0.9909 |  | 117 | 0.9936 |  | 147 | 0.9894 |  | 177 | 0.9927 |
| 28 | 0.9936 |  |  |  |  | 28 | 0.9961 |  | 58 | 0.9930 |  | 88 | 0.9944 |  | 118 | 0.9897 |  | 148 | 0.9954 |  | 178 | 0.9959 |
| 29 | 0.9700 |  |  |  |  | 29 | 0.9950 |  | 59 | 0.9936 |  | 89 | 0.9944 |  | 119 | 0.9947 |  | 149 | 0.9931 |  | 179 | 0.9906 |
| 30 | 0.9910 |  |  |  |  | 30 | 0.9954 |  | 60 | 0.9950 |  | 90 | 0.9941 |  | 120 | 0.9922 |  | 150 | 0.9910 |  |  |  |
