# Supplementary material for: Genome-Wide Association Study Identifies ZNF354C Variants Associated with Depression from Interferon-Based Therapy for Chronic Hepatitis C
Source: PLoS One. 2016 Oct 10;11(10):e0164418. doi: 10.1371/journal.pone.0164418 (PMC5056723; doi:10.1371/journal.pone.0164418)
Supplement: S2 Table — (DOCX) [file pone.0164418.s007.docx]

**S2 Table. Results of replication analysis for 42 SNPs.**

|  |  |  |  | GWAS | | | | | | | |  | Replication | | | | | | | |  | Combined | | | | | | | | |
| --- | --- | --- | --- | --- | --- | --- | --- | --- | --- | --- | --- | --- | --- | --- | --- | --- | --- | --- | --- | --- | --- | --- | --- | --- | --- | --- | --- | --- | --- | --- |
| SNP | dbSNP |  |  |  |  | Case | | | Control | | |  |  |  | Case | | | Control | | |  |  |  | Case | | | Control | | |  |
| Selection ^a^ | rsID | Chr. | Gene | *P*-value | OR | AA | AB | BB | AA | AB | BB |  | *P*-value | OR | AA | AB | BB | AA | AB | BB |  | *P*-value | OR | AA | AB | BB | AA | AB | BB |  |
| 2 | rs7603234 | 2 | SLC9A4 | 7.79E-02 | 1.88 | 36 | 8 | 1 | 117 | 56 | 6 |  | 1.50E-02 | 0.49 | 19 | 17 | 4 | 79 | 38 | 3 |  | 5.87E-01 | 0.89 | 55 | 25 | 5 | 196 | 94 | 9 |  |
| 1 | rs7562732 | 2 | INSIG2 | 9.26E-06 | 0.14 | 36 | 7 | 2 | 172 | 7 | 0 |  | 2.72E-02 | 0.31 | 33 | 7 | 0 | 113 | 7 | 0 |  | 2.05E-06 | 0.20 | 69 | 14 | 2 | 285 | 14 | 0 |  |
| 2 | rs12474475 | 2 | GPD2 | 3.50E-04 | 2.90 | 2 | 17 | 26 | 1 | 32 | 146 |  | 9.20E-01 | 0.96 | 1 | 7 | 32 | 1 | 26 | 93 |  | 9.91E-03 | 1.85 | 3 | 24 | 58 | 2 | 58 | 239 |  |
| 2 | rs12474548 | 2 | GPD2 | 3.27E-04 | 2.60 | 6 | 16 | 23 | 1 | 51 | 127 |  | 8.70E-01 | 0.95 | 2 | 11 | 27 | 8 | 31 | 81 |  | 1.13E-02 | 1.69 | 8 | 27 | 50 | 9 | 82 | 208 |  |
| 1 | rs11886346 | 2 | FIGN | 1.11E-06 | 11.06 | 2 | 6 | 37 | 0 | 4 | 175 |  | 7.52E-01 | 1.21 | 0 | 4 | 36 | 0 | 10 | 110 |  | 2.97E-04 | 3.74 | 2 | 10 | 73 | 0 | 14 | 285 |  |
| 1 | rs1488990 | 3 | TOP2B | 5.01E-05 | 3.06 | 4 | 18 | 23 | 4 | 34 | 141 |  | 8.05E-02 | 1.72 | 1 | 18 | 21 | 5 | 29 | 86 |  | 2.85E-05 | 2.37 | 5 | 36 | 44 | 9 | 63 | 227 |  |
| 2 | rs2364749 | 3 | OXSM | 4.23E-04 | 0.37 | 22 | 18 | 3 | 138 | 37 | 4 |  | 2.04E-02 | 0.50 | 18 | 21 | 1 | 84 | 30 | 5 |  | 2.33E-05 | 0.42 | 40 | 39 | 4 | 222 | 67 | 9 |  |
| 1 | rs4568226 | 4 | RBPJ | 9.64E-07 | 0.14 | 35 | 7 | 3 | 171 | 8 | 0 |  | 5.54E-01 | 1.47 | 37 | 3 | 0 | 107 | 13 | 0 |  | 1.52E-03 | 0.35 | 72 | 10 | 3 | 278 | 21 | 0 |  |
| 1 | rs16877889 | 4 | RBPJ | 2.92E-07 | 0.12 | 35 | 7 | 3 | 172 | 7 | 0 |  | 5.54E-01 | 1.47 | 37 | 3 | 0 | 107 | 13 | 0 |  | 9.58E-04 | 0.33 | 72 | 10 | 3 | 279 | 20 | 0 |  |
| 1 | rs10014388 | 4 | SCD5 | 2.65E-06 | 0.26 | 24 | 15 | 5 | 143 | 31 | 1 |  | 9.79E-01 | 0.99 | 27 | 12 | 1 | 82 | 31 | 5 |  | 7.02E-04 | 0.48 | 51 | 27 | 6 | 225 | 62 | 6 |  |
| 1 | rs16833 | 4 | SCD5 | 3.53E-05 | 3.25 | 4 | 16 | 25 | 2 | 32 | 145 |  | 9.32E-01 | 1.03 | 1 | 12 | 27 | 5 | 31 | 84 |  | 2.25E-03 | 1.95 | 5 | 28 | 52 | 7 | 63 | 229 |  |
| 1 | rs12645341 | 4 | FSTL5 | 2.12E-05 | 2.74 | 10 | 26 | 9 | 13 | 73 | 93 |  | 8.89E-01 | 0.96 | 6 | 12 | 22 | 11 | 52 | 57 |  | 2.45E-03 | 1.72 | 16 | 38 | 31 | 24 | 125 | 150 |  |
| 1 | rs12514360 | 5 | IRX1 | 3.31E-05 | 2.82 | 9 | 17 | 19 | 7 | 52 | 120 |  | 3.83E-01 | 0.76 | 2 | 12 | 26 | 5 | 49 | 65 |  | 1.36E-02 | 1.61 | 11 | 29 | 45 | 12 | 101 | 185 |  |
| 1 | rs16870692 | 5 | C5orf38 | 4.67E-06 | 0.32 | 17 | 20 | 8 | 123 | 49 | 7 |  | 4.44E-01 | 1.27 | 26 | 12 | 2 | 67 | 48 | 5 |  | 4.35E-03 | 0.58 | 43 | 32 | 10 | 190 | 97 | 12 |  |
| 1 | rs347744 | 5 | CDH18 | 2.01E-04 | 0.30 | 29 | 12 | 3 | 153 | 26 | 0 |  | 1.18E-01 | 0.49 | 34 | 6 | 0 | 2 | 30 | 88 |  | 9.16E-03 | 0.50 | 63 | 18 | 3 | 253 | 44 | 1 |  |
| 2 | rs4957930 | 5 | CAMK4 | 1.41E-03 | 2.64 | 33 | 10 | 2 | 80 | 81 | 18 |  | 5.20E-01 | 0.83 | 20 | 15 | 5 | 63 | 48 | 9 |  | 5.19E-02 | 1.48 | 53 | 25 | 7 | 143 | 129 | 27 |  |
| 2 | rs2856354 | 5 | GRM6 | 7.96E-04 | 0.44 | 13 | 28 | 4 | 107 | 63 | 9 |  | 5.01E-01 | 0.82 | 22 | 14 | 4 | 70 | 43 | 7 |  | 3.57E-03 | 0.58 | 35 | 42 | 8 | 177 | 106 | 16 |  |
| 1 | rs4701037 | 5 | ADAMTS2 | 6.99E-06 | 0.34 | 5 | 19 | 21 | 59 | 92 | 28 |  | 9.33E-02 | 0.65 | 7 | 20 | 13 | 38 | 52 | 30 |  | 7.36E-06 | 0.45 | 12 | 39 | 34 | 97 | 144 | 58 |  |
| 1 | rs1562234 | 5 | ADAMTS2 | 6.99E-06 | 0.34 | 5 | 19 | 21 | 59 | 92 | 28 |  | 1.07E-01 | 0.66 | 7 | 20 | 13 | 37 | 53 | 30 |  | 8.84E-06 | 0.46 | 12 | 39 | 34 | 96 | 145 | 58 |  |
| 1 | rs1863918 | 5 | ZNF354C | 2.05E-05 | 2.73 | 12 | 25 | 8 | 15 | 79 | 85 |  | 9.81E-04 | 2.36 | 9 | 23 | 8 | 15 | 44 | 61 |  | 7.89E-08 | 2.55 | 21 | 48 | 16 | 30 | 123 | 146 |  |
| 1 | rs10479525 | 5 | ZNF354C | 6.99E-06 | 2.98 | 21 | 19 | 5 | 29 | 90 | 60 |  | 1.07E-01 | 1.52 | 13 | 20 | 7 | 30 | 53 | 37 |  | 8.84E-06 | 2.19 | 34 | 39 | 12 | 59 | 143 | 97 |  |
| 1 | rs3797590 | 5 | ADAMTS2 | 8.44E-06 | 0.34 | 5 | 20 | 20 | 59 | 95 | 25 |  | 2.79E-02 | 0.57 | 7 | 20 | 13 | 41 | 54 | 25 |  | 1.75E-06 | 0.43 | 12 | 40 | 33 | 100 | 149 | 50 |  |
| 2 | rs2073515 | 6 | ATXN1 | 1.71E-04 | 2.43 | 17 | 18 | 10 | 22 | 85 | 72 |  | 7.40E-01 | 1.09 | 7 | 18 | 15 | 18 | 55 | 47 |  | 2.98E-03 | 1.68 | 24 | 36 | 25 | 40 | 140 | 119 |  |
| 1 | rs9386749 | 6 | --- | 5.10E-07 | 0.31 | 9 | 22 | 14 | 94 | 71 | 14 |  | 2.70E-01 | 0.74 | 15 | 20 | 5 | 57 | 52 | 11 |  | 9.09E-06 | 0.46 | 24 | 42 | 19 | 151 | 123 | 25 |  |
| 1 | rs16930249 | 10 | SVIL | 4.85E-05 | 3.50 | 2 | 16 | 27 | 0 | 27 | 152 |  | 1.41E-01 | 1.90 | 0 | 9 | 31 | 0 | 15 | 105 |  | 6.72E-05 | 2.72 | 2 | 25 | 58 | 0 | 42 | 257 |  |
| 1 | rs11021246 | 11 | FAM76B | 2.54E-05 | 0.30 | 23 | 20 | 2 | 144 | 33 | 1 |  | 9.52E-01 | 0.98 | 30 | 7 | 3 | 83 | 34 | 2 |  | 1.92E-03 | 0.50 | 53 | 27 | 5 | 227 | 67 | 3 |  |
| 1 | rs12361597 | 11 | TMEM123 | 3.87E-05 | 0.33 | 21 | 18 | 5 | 133 | 44 | 2 |  | 9.49E-02 | 0.60 | 21 | 17 | 2 | 80 | 36 | 3 |  | 2.99E-05 | 0.43 | 42 | 35 | 7 | 213 | 80 | 5 |  |
| 1 | rs11225264 | 11 | MMP7 | 2.35E-05 | 0.33 | 21 | 19 | 5 | 133 | 44 | 2 |  | 1.27E-01 | 0.63 | 21 | 17 | 2 | 79 | 38 | 3 |  | 3.22E-05 | 0.44 | 42 | 36 | 7 | 212 | 82 | 5 |  |
| 1 | rs12428431 | 13 | RFC3 | 2.28E-05 | 4.31 | 37 | 7 | 1 | 79 | 84 | 16 |  | 1.00E+00 | 1.00 | 23 | 15 | 2 | 69 | 45 | 6 |  | 1.11E-03 | 2.06 | 60 | 22 | 3 | 148 | 129 | 22 |  |
| 1 | rs1508486 | 13 | NBEA | 7.96E-04 | 2.28 | 7 | 22 | 16 | 21 | 39 | 119 |  | 7.85E-01 | 0.93 | 6 | 14 | 20 | 21 | 40 | 59 |  | 1.99E-02 | 1.53 | 13 | 36 | 36 | 42 | 79 | 178 |  |
| 1 | rs9564594 | 13 | MAB21L1 | 1.09E-04 | 2.66 | 4 | 25 | 16 | 4 | 56 | 119 |  | 8.61E-01 | 1.05 | 2 | 18 | 20 | 4 | 54 | 59 |  | 2.80E-03 | 1.77 | 6 | 43 | 36 | 8 | 110 | 178 |  |
| 1 | rs17488796 | 14 | NPAS3 | 2.28E-05 | 0.30 | 24 | 18 | 3 | 147 | 29 | 3 |  | 1.62E-01 | 1.65 | 30 | 9 | 1 | 75 | 40 | 5 |  | 4.34E-02 | 0.64 | 54 | 27 | 4 | 222 | 69 | 8 |  |
| 2 | rs8020629 | 14 | NRXN3 | 8.16E-04 | 0.34 | 30 | 13 | 2 | 154 | 24 | 1 |  | 9.17E-02 | 0.51 | 30 | 9 | 1 | 103 | 16 | 1 |  | 3.22E-04 | 0.40 | 60 | 22 | 3 | 257 | 40 | 2 |  |
| 2 | rs759233 | 14 | NRXN3 | 8.16E-04 | 2.97 | 2 | 13 | 30 | 1 | 24 | 154 |  | 9.17E-02 | 1.97 | 1 | 9 | 30 | 1 | 16 | 103 |  | 3.22E-04 | 2.48 | 3 | 22 | 60 | 2 | 40 | 257 |  |
| 1 | rs4904887 | 14 | SLC24A4 | 8.55E-05 | 0.39 | 13 | 23 | 9 | 102 | 66 | 11 |  | 1.70E-03 | 2.83 | 28 | 12 | 0 | 55 | 50 | 15 |  | 4.33E-01 | 0.86 | 41 | 35 | 9 | 157 | 116 | 26 |  |
| 1 | rs7176464 | 15 | RGMA | 7.37E-05 | 0.27 | 29 | 15 | 1 | 158 | 21 | 0 |  | 2.58E-01 | 1.87 | 35 | 4 | 0 | 98 | 22 | 0 |  | 2.80E-02 | 0.54 | 64 | 19 | 1 | 256 | 43 | 0 |  |
| 1 | rs7190922 | 16 | FLJ11151 | 2.43E-05 | 2.80 | 7 | 24 | 14 | 6 | 62 | 111 |  | 1.00E+00 | 1.00 | 2 | 12 | 26 | 6 | 36 | 78 |  | 1.87E-03 | 1.82 | 9 | 36 | 40 | 12 | 98 | 189 |  |
| 1 | rs2779180 | 19 | M6PRBP1 | 1.02E-04 | 0.36 | 20 | 20 | 5 | 128 | 47 | 4 |  | 3.68E-03 | 0.43 | 20 | 15 | 5 | 87 | 27 | 6 |  | 1.30E-06 | 0.39 | 40 | 35 | 10 | 215 | 74 | 10 |  |
| 1 | rs1609997 | 20 | STK35 | 7.86E-05 | 3.37 | 2 | 16 | 27 | 0 | 28 | 151 |  | 3.12E-01 | 0.60 | 0 | 5 | 35 | 1 | 22 | 97 |  | 2.13E-02 | 1.81 | 2 | 21 | 62 | 1 | 50 | 248 |  |
| 1 | rs4813398 | 20 | TGM3 | 3.40E-05 | 3.41 | 2 | 18 | 25 | 1 | 29 | 149 |  | 3.40E-01 | 0.64 | 0 | 6 | 34 | 1 | 25 | 94 |  | 1.35E-02 | 1.84 | 2 | 24 | 59 | 2 | 54 | 243 |  |
| 2 | rs2870630 | 20 | CBLN4 | 1.00E-03 | 0.45 | 8 | 30 | 6 | 90 | 73 | 16 |  | 5.94E-01 | 1.15 | 17 | 18 | 5 | 41 | 66 | 13 |  | 3.60E-02 | 0.69 | 25 | 48 | 11 | 131 | 139 | 29 |  |
| 2 | rs2426580 | 20 | CBLN4 | 3.36E-04 | 2.56 | 4 | 21 | 20 | 3 | 50 | 126 |  | 5.51E-01 | 0.83 | 1 | 16 | 23 | 7 | 48 | 65 |  | 2.66E-02 | 1.55 | 5 | 37 | 43 | 10 | 98 | 191 |  |

SNP, single nucleotide polymorphism; Chr, chromosome; GWAS, genome-wide association study; OR, odds ratio.

*P*-value by the chi-square test for the allele frequency model. OR for the allele frequency model. Data of subjects whose genotypes were not determined were excluded.

^a^ For SNP selection, "1" included 31 SNPs with *P*<10^-4^ at GWAS, "2" included 11 SNPs that were considered candidate genetic regions and added to the replication study (not included in GWAS). The results of GWAS and replication stage for 42 SNPs were obtained by genotyping using the DigiTag2 assay.
