# Supplementary material for: Genome-Wide Association Study Identifies ZNF354C Variants Associated with Depression from Interferon-Based Therapy for Chronic Hepatitis C
Source: PLoS One. 2016 Oct 10;11(10):e0164418. doi: 10.1371/journal.pone.0164418 (PMC5056723; doi:10.1371/journal.pone.0164418)
Supplement: S3 Table — (DOCX) [file pone.0164418.s008.docx]

**S3 Table. Further details of the 11 SNPs used for replication analysis.**

| dbSNP  rsID | Nearest  gene | Chr. | Physical Position  (dbSNP137) | Associated diseases or trait | References #. |
| --- | --- | --- | --- | --- | --- |
| rs7603234 | SLC9A4 | 2 | 102592980 | white blood cell subtypes, celiac disease related to the immune response, IL-33/ST2 signaling | [1-3] |
| rs12474475 | GPD2 | 2 | 157582110 | ulcerative colitis, lipid-lowering response to statins | [4, 5] |
| rs12474548 | GPD2 | 2 | 157582316 | ulcerative colitis, lipid-lowering response to statins | [4, 5] |
| rs2364749 | OXSM | 3 | 25816531 | AIDS progression | [6] |
| rs4957930 | CAMK4 | 5 | 110560808 | Alzheimer's disease, allergic sensitization, blood pressure and arterial stiffness | [7-9] |
| rs2856354 | GRM6 | 5 | 178338335 | pubertal height growth, pubertal timing and childhood adiposity | [10] |
| rs2073515 | ATXN1 | 6 | 16767140 | antidepressant efficacy in major depressive disorder | [11] |
| rs8020629 | NRXN3 | 14 | 79336370 | obesity, performance on standardized cognitive tests, neurodegeneration in Alzheimer's disease | [12-14] |
| rs759233 | NRXN3 | 14 | 79340786 | obesity, performance on standardized cognitive tests, neurodegeneration in Alzheimer's disease | [12-14] |
| rs2870630 | CBLN4 | 20 | 53512864 | multiple signaling pathways, preferentially inhibitory presynaptic differentiation of cortical neurons | [15, 16] |
| rs2426580 | CBLN4 | 20 | 53518087 | multiple signaling pathways, preferentially inhibitory presynaptic differentiation of cortical neurons | [15, 16] |

SNP, single nucleotide polymorphism; Chr, chromosome; GWAS, genome-wide association study.

**References**

1. Okada Y, Hirota T, Kamatani Y, Takahashi A, Ohmiya H, Kumasaka N, et al. Identification of nine novel loci associated with white blood cell subtypes in a Japanese population. PLoS genetics. 2011;7(6):e1002067.

2. Hunt KA, Zhernakova A, Turner G, Heap GA, Franke L, Bruinenberg M, et al. Newly identified genetic risk variants for celiac disease related to the immune response. Nat Genet. 2008;40(4):395-402.

3. Ho JE, Chen WY, Chen MH, Larson MG, McCabe EL, Cheng S, et al. Common genetic variation at the IL1RL1 locus regulates IL-33/ST2 signaling. The Journal of clinical investigation. 2013;123(10):4208-18.

4. Julia A, Domenech E, Chaparro M, Garcia-Sanchez V, Gomollon F, Panes J, et al. A genome-wide association study identifies a novel locus at 6q22.1 associated with ulcerative colitis. Hum Mol Genet. 2014;23(25):6927-34.

5. Barber MJ, Mangravite LM, Hyde CL, Chasman DI, Smith JD, McCarty CA, et al. Genome-wide association of lipid-lowering response to statins in combined study populations. PloS one. 2010;5(3):e9763.

6. Hendrickson SL, Lautenberger JA, Chinn LW, Malasky M, Sezgin E, Kingsley LA, et al. Genetic variants in nuclear-encoded mitochondrial genes influence AIDS progression. PloS one. 2010;5(9):e12862.

7. Sherva R, Tripodis Y, Bennett DA, Chibnik LB, Crane PK, de Jager PL, et al. Genome-wide association study of the rate of cognitive decline in Alzheimer's disease. Alzheimer's & dementia : the journal of the Alzheimer's Association. 2014;10(1):45-52.

8. Bonnelykke K, Matheson MC, Pers TH, Granell R, Strachan DP, Alves AC, et al. Meta-analysis of genome-wide association studies identifies ten loci influencing allergic sensitization. Nat Genet. 2013;45(8):902-6.

9. Levy D, Larson MG, Benjamin EJ, Newton-Cheh C, Wang TJ, Hwang SJ, et al. Framingham Heart Study 100K Project: genome-wide associations for blood pressure and arterial stiffness. BMC medical genetics. 2007;8 Suppl 1:S3.

10. Cousminer DL, Berry DJ, Timpson NJ, Ang W, Thiering E, Byrne EM, et al. Genome-wide association and longitudinal analyses reveal genetic loci linking pubertal height growth, pubertal timing and childhood adiposity. Hum Mol Genet. 2013;22(13):2735-47.

11. Common genetic variation and antidepressant efficacy in major depressive disorder: a meta-analysis of three genome-wide pharmacogenetic studies. The American journal of psychiatry. 2013;170(2):207-17.

12. Wang K, Li WD, Zhang CK, Wang Z, Glessner JT, Grant SF, et al. A genome-wide association study on obesity and obesity-related traits. PloS one. 2011;6(4):e18939.

13. Cirulli ET, Kasperaviciute D, Attix DK, Need AC, Ge D, Gibson G, et al. Common genetic variation and performance on standardized cognitive tests. European journal of human genetics : EJHG. 2010;18(7):815-20.

14. Stein JL, Hua X, Morra JH, Lee S, Hibar DP, Ho AJ, et al. Genome-wide analysis reveals novel genes influencing temporal lobe structure with relevance to neurodegeneration in Alzheimer's disease. NeuroImage. 2010;51(2):542-54.

15. Wei P, Pattarini R, Rong Y, Guo H, Bansal PK, Kusnoor SV, et al. The Cbln family of proteins interact with multiple signaling pathways. Journal of neurochemistry. 2012;121(5):717-29.

16. Yasumura M, Yoshida T, Lee SJ, Uemura T, Joo JY, Mishina M. Glutamate receptor delta1 induces preferentially inhibitory presynaptic differentiation of cortical neurons by interacting with neurexins through cerebellin precursor protein subtypes. Journal of neurochemistry. 2012;121(5):705-16.
